# Mechanistic Drivers of Müllerian Duct Development and Differentiation Into the Oviduct

**DOI:** 10.3389/fcell.2021.605301

**Published:** 2021-03-08

**Authors:** Laura Santana Gonzalez, Ioanna A. Rota, Mara Artibani, Matteo Morotti, Zhiyuan Hu, Nina Wietek, Abdulkhaliq Alsaadi, Ashwag Albukhari, Tatjana Sauka-Spengler, Ahmed A. Ahmed

**Affiliations:** ^1^MRC Weatherall Institute of Molecular Medicine, University of Oxford, Oxford, United Kingdom; ^2^Nuffield Department of Women’s and Reproductive Health, University of Oxford, Oxford, United Kingdom; ^3^Developmental Immunology Research Group, Department of Paediatrics, University of Oxford, Oxford, United Kingdom; ^4^Gene Regulatory Networks in Development and Disease Laboratory, MRC Weatherall Institute of Molecular Medicine, Radcliffe Department of Medicine, University of Oxford, Oxford, United Kingdom; ^5^Department of Biochemistry, Faculty of Science, King Abdulaziz University, Jeddah, Saudi Arabia

**Keywords:** Müllerian ducts, oviducts, embryonic development, differentiation, gene regulatory networks, epithelial-mesenchymal transition (EMT), genome-wide survey

## Abstract

The conduits of life; the animal oviducts and human fallopian tubes are of paramount importance for reproduction in amniotes. They connect the ovary with the uterus and are essential for fertility. They provide the appropriate environment for gamete maintenance, fertilization and preimplantation embryonic development. However, serious pathologies, such as ectopic pregnancy, malignancy and severe infections, occur in the oviducts. They can have drastic effects on fertility, and some are life-threatening. Despite the crucial importance of the oviducts in life, relatively little is known about the molecular drivers underpinning the embryonic development of their precursor structures, the Müllerian ducts, and their successive differentiation and maturation. The Müllerian ducts are simple rudimentary tubes comprised of an epithelial lumen surrounded by a mesenchymal layer. They differentiate into most of the adult female reproductive tract (FRT). The earliest sign of Müllerian duct formation is the thickening of the anterior mesonephric coelomic epithelium to form a placode of two distinct progenitor cells. It is proposed that one subset of progenitor cells undergoes partial epithelial-mesenchymal transition (pEMT), differentiating into immature Müllerian luminal cells, and another subset undergoes complete EMT to become Müllerian mesenchymal cells. These cells invaginate and proliferate forming the Müllerian ducts. Subsequently, pEMT would be reversed to generate differentiated epithelial cells lining the fully formed Müllerian lumen. The anterior Müllerian epithelial cells further specialize into the oviduct epithelial subtypes. This review highlights the key established molecular and genetic determinants of the processes involved in Müllerian duct development and the differentiation of its upper segment into oviducts. Furthermore, an extensive genome-wide survey of mouse knockout lines displaying Müllerian or oviduct phenotypes was undertaken. In addition to widely established genetic determinants of Müllerian duct development, our search has identified surprising associations between loss-of-function of several genes and high-penetrance abnormalities in the Müllerian duct and/or oviducts. Remarkably, these associations have not been investigated in any detail. Finally, we discuss future directions for research on Müllerian duct development and oviducts.

## Introduction

The paramesonephric or Müllerian ducts are the embryonic anlagen of most of the female reproductive tract. They give rise to the oviducts (human fallopian tubes), uterine horns (human uterus and chicken shell gland), cervix and upper vagina, in an anterior-to-posterior fashion. The mammalian FRT is of paramount importance in female biology due to its role in promoting a successful fertilization and pregnancy. Concretely, the oviducts provide the environment for gametes nutrition and transport, fertilization and the development and transport of the preimplantation blastocyst (Killian, 2004; Li and Winuthayanon, 2017). Later processes such as blastocyst implantation and fetal development occur in the uterine horns (Kim and Kim, 2017). The reproductive pattern is similar in chickens (Bellairs and Osmond, 2005). Furthermore, the FRT is the site of origin of serious human diseases such as ovarian (George et al., 2016; Zhang et al., 2019), endometrial (Bansal et al., 2009; Morice et al., 2016) and cervical cancer (Cohen et al., 2019); ectopic pregnancy (Rana et al., 2013); and pelvic inflammatory diseases such as endometriosis (Zondervan et al., 2018), salpingitis (Owhor et al., 2019), and hydrosalpinx (Ng and Cheong, 2019). Many of these diseases are life-threatening and can cause infertility.

Despite the crucial importance of this system in female life and disease, reproductive adult organs and the molecular mechanisms underlying their homeostasis, hormonal cycles, pregnancy and associated diseases have been poorly characterized. The lack of knowledge of developmental processes orchestrating FRT formation is, among other reasons, a causative factor and an increasing interest in the field has been detected in recent years (Mullen and Behringer, 2014; Cunha et al., 2018; Roly et al., 2018; Zhao and Yao, 2019). To address this gap, Müllerian duct development is reviewed in this work. A special focus is placed on the anterior Müllerian duct portion differentiating into oviducts, as they are the least studied of the FRT organs and yet are the site of origin of high-grade serous ovarian cancer (George et al., 2016; Zhang et al., 2019), the deadliest gynecological malignancy; tubal causes of infertility; ectopic pregnancy (Rana et al., 2013); and pelvic inflammatory disease (Ng and Cheong, 2019; Owhor et al., 2019).

During embryonic development, the intermediate mesoderm of the gastrula gives rise to two urogenital mesonephroi that will host the female (Müllerian) and male (Wolffian) primitive genital tracts. These reproductive tubes are essential during embryonic sex determination and both ducts provisionally develop regardless of the genetic sex (female: mammal XX and avian ZW; male: mammal XY and avian ZZ). The Wolffian or mesonephric ducts originate directly from the intermediate mesoderm, followed by Müllerian duct development from the anterior mesonephric coelomic epithelium (Gruenwald, 1941; Jacob et al., 1999; Guioli et al., 2007; Orvis and Behringer, 2007; Yoshino et al., 2014; Atsuta and Takahashi, 2016). In XY/ZZ embryos, Wolffian ducts are maintained and Müllerian ducts regress, and vice versa in XX/ZW embryos. These mechanisms are conserved among species, except in class Aves females, where the right gonad and Müllerian duct additionally regress whereas the left equivalents become fully functional (Gruenwald, 1941; Dohr and Tarmann, 1984; Jacob et al., 1999; Guioli et al., 2007; Orvis and Behringer, 2007; Atsuta and Takahashi, 2016). Müllerian ducts are histomorphologically similar during ontogeny and birth, comprising a simple columnar luminal epithelium surrounded by mesenchymal stroma (Komatsu and Fujita, 1978; Yamanouchi et al., 2010; Ghosh et al., 2017). Although certain degree of differentiation is accomplished during this early life period, it is only during the first 2 weeks of life that the primitive tube undergoes an intense morphological change governed by genetic programmes in which every Müllerian section terminally differentiates into an adult reproductive organ (Cunha, 1976; Komatsu and Fujita, 1978; Kurita et al., 2001; Yamanouchi et al., 2010; Ghosh et al., 2017).

The scope of this review focuses on the cellular mechanisms and genetic/molecular entities involved in Müllerian development and successive differentiation into oviducts. This review is structured in two sections: (1) the chronological three-step Müllerian development and oviduct differentiation as well as (2) advanced insights into the Müllerian genetics and developmental pathways. These developmental genetic families mainly comprise master embryonic transcriptional regulators in the homeobox and paired box families, secreted proteins in the WNT [wingless-type mouse mammary tumor virus (MMTV) integration site family] family, RARs (retinoic acid receptors), cofactors, kinases and a G protein-coupled receptor. The bone morphogenic protein (BMP), WNT, transforming growth factor beta (TFG-β), phosphatidylinositol 3-kinase (PI3K)/protein kinase B (Akt) pathway, G-protein coupled receptor and fibroblast growth factor (FGF) signaling pathways are also involved in Müllerian/oviduct biology. Furthermore, literature on cell populations forming the Müllerian ducts is discussed and novel theories on cell lineages, hierarchies and dynamics throughout Müllerian and oviduct life are proposed. This review also includes a thorough genome-wide interrogation of knockout mouse databases searching Müllerian/oviduct phenotypes, which identified new candidate genes that might play an important role in their biology.

## Sections

### Müllerian Ducts Originate From the Mesonephric Coelomic Epithelium, Remain in XX/ZW Embryos and Anteriorly Differentiate Into the Oviducts

**FIGURE 1:**
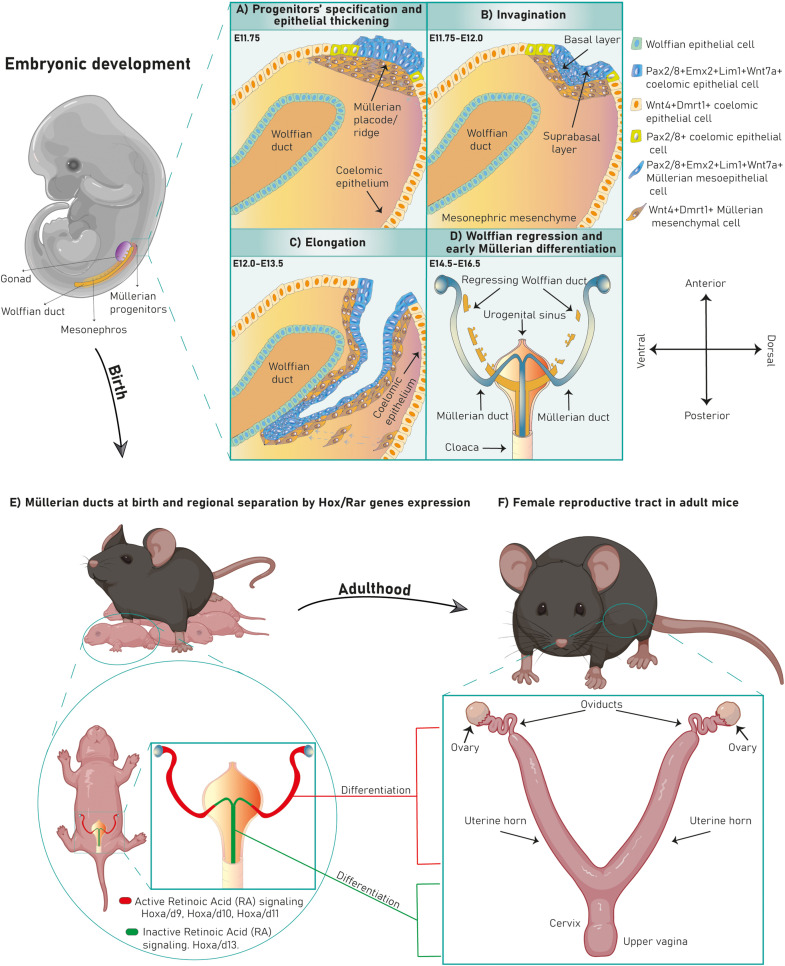
Murine FRT development. Mouse Müllerian ducts originate between E11.75–13.5. The first developmental phase consists of Müllerian progenitors’ specification and placode formation in the mesonephric coelomic epithelium. Pax2^+^/8^+^/Emx2^+^/Lim1^+^/Wnt7a^+^ epithelial cells (mesoepithelial progenitors) give rise to the tubal lumen whereas Wnt4^+^/Dmrt1^+^ epithelial cells are mesenchymal progenitors **(A)**. Subsequently, epithelial invagination occurs through apical constriction of suprabasal epithelial cells in the placode, ending when the proliferative Müllerian tip establishes physical contact with the Wolffian duct at E12.0 **(B)**. Afterward, the Müllerian ducts elongate following the anterior–posterior axis until E13.5 **(C)**. Finally, the Wolffian ducts regress **(D)** and the Müllerian ducts undertake an initial regional differentiation marked by a Hox/RAR axis **(E)**. During the first 2 weeks of life, every FRT section completely differentiates, giving rise to the functional female reproductive tract **(F)**.

Embryonic Müllerian development is highly conserved among avian and mammalian species and it is executed in three phases (Gruenwald, 1941; Dohr and Tarmann, 1984; Jacob et al., 1999; Guioli et al., 2007; Orvis and Behringer, 2007; Atsuta and Takahashi, 2016) (Figures 1A–C). In mouse it occurs between E11.75-13.5 (Kobayashi et al., 2004; Guioli et al., 2007; Orvis and Behringer, 2007), in human between Carnegie stages 18–23 (Hashimoto, 2003) and in chicken between HH16-HH30/32 (Guioli et al., 2007; Atsuta and Takahashi, 2016). After Müllerian development, the Wolffian ducts regress in females (Figure 1D) and the Müllerian ducts differentiate into the adult FRT (Figures 1E,F).

#### Embryonic Development of Müllerian Ducts: Cellular and Molecular Mechanisms

**FIGURE 2:**
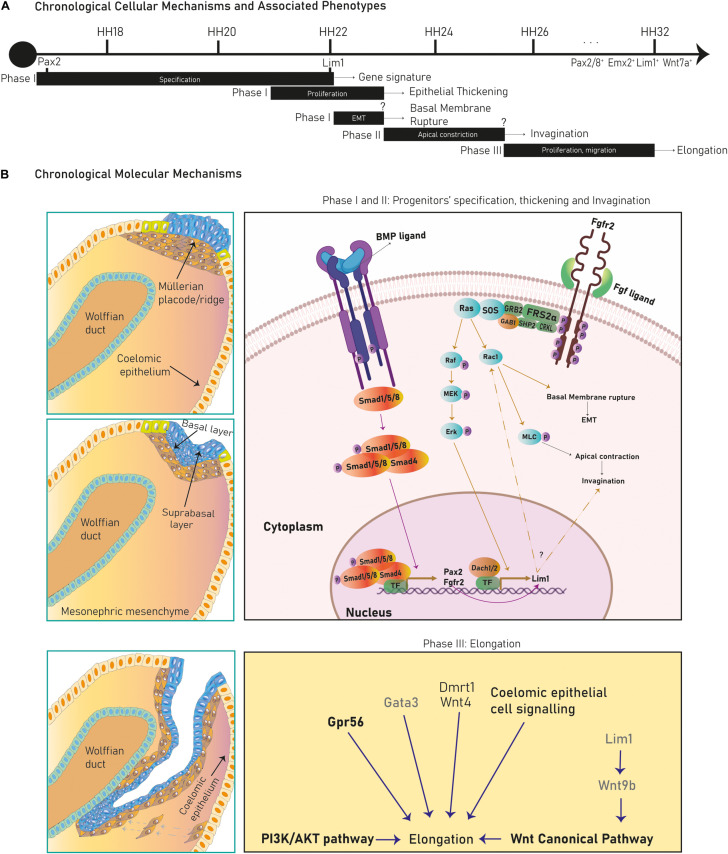
Cellular mechanisms and molecular pathways in Müllerian duct development. Several cellular mechanisms chronologically succeed during chicken Müllerian duct development **(A)**. In **(B)**, early stages in Müllerian duct development are governed by the BMP pathway regulating Pax2, activating the FGF/RAS/ERK pathway and promoting Lim1 expression and apical constriction control. Dach1/2 and Pax2 also regulate Lim1. Later, development proceeds through rostral-caudal elongation depending on Wolffian paracrine signals, such as Lim1-regulated Wnt9b, which activates the Wnt Canonical pathway. Besides, the PI3k/Akt pathway, coelomic epithelium signaling and Wnt4, Gata3, Gpr56, and Dmrt1 regulate elongation. Genes/pathways in bold are expressed/intrinsic to Müllerian epithelial cells, genes in light are expressed by mesonephric mesenchymal cells and genes in gray are expressed by Wolffian epithelial cells.

The Müllerian ducts develop from the anterior mesonephric coelomic epithelium and elongate along the anteroposterior axis between the Wolffian duct and the coelomic epithelium. After elongation they fuse with the urogenital sinus and differentiate into the FRT. The Müllerian developmental process is divided into three stages marked by diverse cellular and molecular mechanisms (Figure 2A).

##### Phase I: progenitors’ specification and epithelial thickening

**FIGURE 3:**
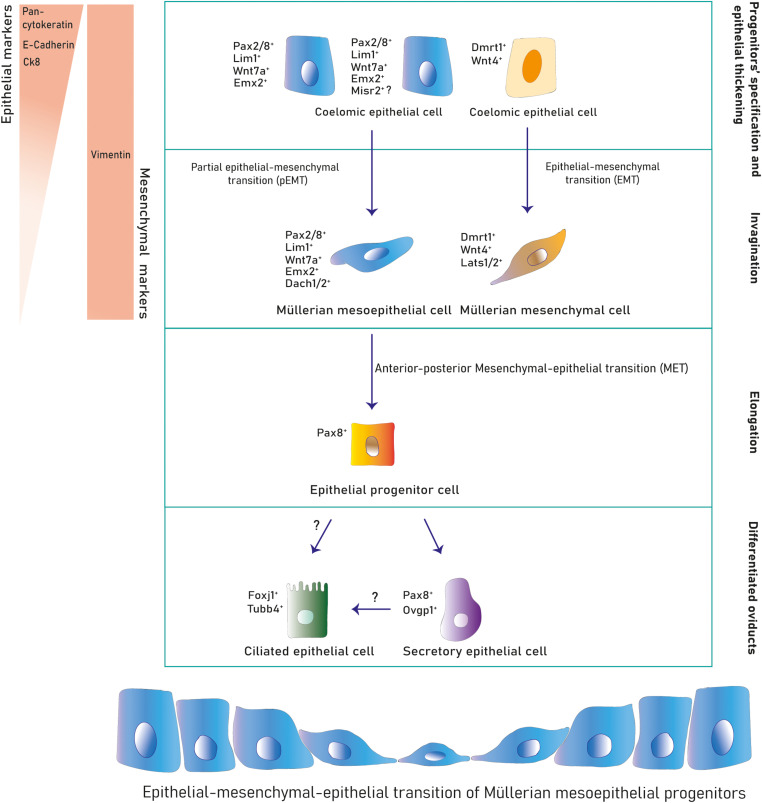
Cell lineages during Müllerian development and oviduct differentiation. Two primitive populations expressing distinct markers in the mesonephric coelomic epithelium give rise to the Müllerian epithelial and mesenchymal lineages. Mesoepithelial progenitors execute a pEMT into the mesoepithelial type forming the lumen and mesenchymal progenitors undergo EMT differentiating into mesenchyme. After Müllerian duct formation, the mesoepithelium reverses to an epithelial identity that differentiates into secretory and ciliated oviduct epithelial subtypes postnatally.

During this stage, cells from the cranial coelomic epithelial surface on both mesonephroi undertake fate specification into Müllerian progenitors, and a placode/ridge forms close to the Wolffian duct (Gruenwald, 1941, Dohr and Tarmann, 1984, Jacob et al., 1999, Kobayashi et al., 2004, Guioli et al., 2007, Orvis and Behringer, 2007 and Atsuta and Takahashi, 2016) (Figure 1A). This process is conserved across most species. A placode is a multi-layered epithelial thickening forming basal and supra-basal strata of cells and created by cell proliferation (Figure 1B). The Müllerian ridge contains Müllerian mesenchymal and mesoepithelial progenitors and BMP signaling is essential for its development in chicken embryos (Atsuta and Takahashi, 2016). Moreover, an independent bulk-RNA study on chicken Müllerian ducts found that genes related to positive regulation of pathway-restricted SMAD protein phosphorylation and SMAD protein signal transduction were enriched at this stage, supporting a role for the BMP pathway (Roly et al., 2020b). The BMP effector molecule/s is currently unknown, although expression of Bmp2/3/4/7 has been documented during chicken Müllerian development (Atsuta and Takahashi, 2016; Roly et al., 2020b). Regarding Müllerian progenitors, the term “mesoepithelial” is adopted across species as these progenitors give rise to cells negative for epithelial markers (negative: Ck8 and *E*-cadherin; weakly positive: pan-cytokeratin) and positive for a mesenchymal marker (vimentin) while showing mesenchymal morphology and arrangement upon the basement membrane (BM), which is an epithelial feature (Gruenwald, 1941; Dohr and Tarmann, 1984; Jacob et al., 1999; Orvis and Behringer, 2007; Prunskaite-Hyyryläinen et al., 2016; Ghosh et al., 2020; Roly et al., 2020a). On the other hand, mesenchymal progenitors differentiate into cells of a true mesenchymal phenotype. This suggests that mesoepithelial progenitors undergo partial epithelial-mesenchymal transition (pEMT), whereas mesenchymal progenitors fully complete an EMT (Figure 3). The first EMT step observed in this context is the rupture of the placode BM, positively regulated by the FGF pathway in chickens. When the FGF receptor *Fgfr2* and the intracellular modulator *Rac1* are downregulated, the placode BM fails to break (Atsuta and Takahashi, 2016) (Figure 2B). The role of the FGF/ERK (extracellular signal-related kinase)/MAPK (mitogen-activated protein kinase) pathway is reaffirmed due to the expression of several pathway genes during chicken Müllerian development (Roly et al., 2020b). The FGF molecular regulator is unknown, although upregulated expression of *Fgf10/16*/*18* has been reported during chicken Müllerian development (Roly et al., 2020b). Interestingly, the BMP pathway positively regulates the FGF/RAS/MAPK pathway in chicken Müllerian ducts (Atsuta and Takahashi, 2016).

The murine Müllerian luminal epithelium is exclusively derived from mesoepithelial progenitors in the placode expanding along the anterior–posterior axis (Ghosh et al., 2020). Conversely, in mouse and chicken, mesenchymal cells derive from progenitors in the placode migrating anteroposteriorly and from progenitors along the coelomic mesonephric epithelium undergoing local delamination and dorsal-ventral migration (Guioli et al., 2007; Ayers et al., 2015; Prunskaite-Hyyryläinen et al., 2016) (Figures 1A–C, 3). Mesoepithelial progenitors sequentially upregulate the embryonic transcription factors *Pax2*, *Emx2*, and *Lim1* (also *Pax8, Pbx1, Hnf1b*, and *Wnt7a* in mouse), while mesenchymal precursors express markers such as *Wnt4* (and *Dmrt1* in chicken) (Jacob et al., 1999; Vainio et al., 1999; Schnabel et al., 2003; Kobayashi et al., 2004; Guioli et al., 2007; Orvis and Behringer, 2007; Lokmane et al., 2010; Ayers et al., 2015; Atsuta and Takahashi, 2016; Prunskaite-Hyyryläinen et al., 2016). Expression of the Dach1/2 transcriptional cofactors has also been reported in murine mesoepithelial progenitors (Davis et al., 2008). Some mouse mesoepithelial progenitors have been shown to express *Misr2*, although this putative subpopulation has not been further studied (Ghosh et al., 2020).

In chicken mesoepithelial progenitors, *Pax2* expression is regulated by the BMP pathway (Figure 2B) and controls *Lim1* expression (Atsuta and Takahashi, 2016). *Lim1* expression is also governed by the FGF/ERK/MAPK pathway as cell electroporation with a dominant-negative *Fgfr2* and Ras inhibition decreased phosphorylated ERK (pERK) levels and abolished *Lim1* expression (Atsuta and Takahashi, 2016) (Figure 2B). Additionally, *Lim1* expression in chickens requires unknown Wolffian duct signals (Atsuta and Takahashi, 2016), although this specification is not clear in mammals.

##### Phase II: invagination

After progenitor specification and placode formation, *Wnt4^+^/Dmrt1^+^* progenitors differentiate into mesenchymal cells, whereas *Pax2^+^/Emx2^+^/Lim1^+^* progenitors give rise to mesoepithelial cells (Figures 1B, 3). They invaginate until the growing duct tip establishes physical contact with the Wolffian duct (Gruenwald, 1941; Dohr and Tarmann, 1984; Jacob et al., 1999; Hashimoto, 2003; Kobayashi et al., 2004; Guioli et al., 2007; Orvis and Behringer, 2007). The cellular mechanism behind Müllerian epithelial invagination is cell intercalation-mediated apical contraction of supra-basal cells (Atsuta and Takahashi, 2016; Pearl et al., 2017), which transmits tension to the BM, resulting in bending. Apical constriction is executed by an actomyosin skeleton of F-actin filaments and non-muscle myosin II, a motor protein activated by its phosphorylated myosin light chain (pMLC) (Sellers, 1991; Bresnick, 1999; Martin and Goldstein, 2014) (Figure 2B). pMLC is apically detected in chicken *Pax2^+^/Lim1^+^* cells before Müllerian invagination (Atsuta and Takahashi, 2016). *Fgfr2* and *Rac1* downregulation decreases pMLC and disrupts invagination, indicating a possible role for FGF signaling in chicken (Atsuta and Takahashi, 2016) (Figure 2B). Additionally, the PI3K/Akt pathway is activated at this stage in rodents although its specific role has not been studied (Fujino et al., 2009). Müllerian invagination is controlled by Lim1 and Wnt4, expressed by mesoepithelial and mesenchymal progenitors respectively (Vainio et al., 1999; Kobayashi et al., 2004; Orvis and Behringer, 2007; Atsuta and Takahashi, 2016). In *Lim1*-null and *Wnt4*-null knock-out mice, epithelial invagination is blocked, although *Lim1^+^/Wnt7a^+^* progenitors are detected in *Wnt4*-nulls suggesting that Wnt4 does not control mesoepithelial progenitors’ specification and that, likely, a two-axes mesenchymal-epithelial signaling is required for invagination (Kobayashi and Behringer, 2003; Kobayashi et al., 2004; Guioli et al., 2007; Prunskaite-Hyyryläinen et al., 2016). In chicken embryos, loss of Lim1 also blocks invagination (Atsuta and Takahashi, 2016).

##### Phase III: caudal elongation

Finally, the Müllerian architecture is achieved by two-dimensional growth. First, the developing Müllerian tip undergoes caudal elongation along the anteroposterior axis through apical constriction, cell proliferation and cranial-caudal cell migration (Gruenwald, 1941; Jacob et al., 1999; Kobayashi et al., 2004; Guioli et al., 2007; Orvis and Behringer, 2007; Fujino et al., 2009; Atsuta and Takahashi, 2016). Second, the deposited cells proliferate to expand the tube on the dorsoventral axis (Jacob et al., 1999; Guioli et al., 2007; Orvis and Behringer, 2007) (Figure 1C). Initially, the BM is shared between the Wolffian ducts and the Müllerian mesoepithelial tip. Subsequently, as the Müllerian duct elongates, the number of mesenchymal cells between the ducts increases, establishing a physical separation regulated by PI3k in rodents, which splits the female and male BMs (Gruenwald, 1941; Jacob et al., 1999; Hashimoto, 2003; Fujino et al., 2009). However, the Wolffian ducts remain in physical contact with the Müllerian proliferative tip, guiding and supporting its growth during the whole process, although Wolffian ducts do not contribute cells to the paramesonephric ducts (Gruenwald, 1941; Dohr and Tarmann, 1984; Hashimoto, 2003; Carroll et al., 2005; Kobayashi et al., 2005; Guioli et al., 2007; Orvis and Behringer, 2007). Murine Müllerian elongation depends on physical and *Wnt9b*- and *Gata3*-mediated paracrine interactions with the Wolffian duct. Both genes are expressed by the Wolffian epithelium. In mouse, Wnt9b controls Müllerian elongation through the canonical WNT pathway and the loss of Gata3 prompts elongation arrest (Carroll et al., 2005; Grote et al., 2008). Elongation also depends on the Gpr56 receptor expressed in the mesoepithelial progenitors (chicken), the mesenchymal expression of *Wnt4* and *Dmrt1* (chicken), the PI3K/Akt pathway (rodents) and coelomic epithelium-derived paracrine signals (chicken) (Gruenwald, 1941; Carroll et al., 2005; Kobayashi et al., 2005; Pedersen et al., 2005; Fujino et al., 2009; Atsuta and Takahashi, 2016; Prunskaite-Hyyryläinen et al., 2016; Roly et al., 2020a) (Figure 2B). This crosstalk between the coelomic epithelium and the elongating tube is evident, given that *Wnt4* expression is activated along the chicken coelomic epithelium as elongation advances and that physical disruption of the coelomic epithelium blocks Müllerian development at the mechanical incision level (Fujino et al., 2009; Prunskaite-Hyyryläinen et al., 2016).

Overall, Müllerian duct development is a complex three-stage process encompassing key cellular mechanisms and its successful execution requires the coordinated action of several developmental pathways and genetic entities. Recently, a high-throughput bulk-RNA sequencing study of developing chicken Müllerian ducts, confirmed the aforementioned cellular mechanisms (Roly et al., 2020b). In this study, genes related to transcription and cell differentiation were differentially expressed during the early and the last stages of development, coinciding with the progenitors’ specification and the initial post-developmental differentiation respectively. Cell migration and negative regulation of apoptosis were upregulated at elongation whereas cell adhesion and cell proliferation mechanisms were enriched during all the developmental stages. After Müllerian development, the ducts merge on their caudal end and with the endoderm-derived urogenital sinus. It has been demonstrated that a transmembrane protein of unknown function, Lhfpl2 (lipoma HMGIC fusion partner-like 2), is behind the scenes as a point mutation in this gene prevents murine Müllerian tips to fuse with the urogenital sinus (Zhao et al., 2016).

#### Sex-Specified Wolffian Duct Regression, Müllerian Duct Maintenance and Anterior Differentiation Into Oviducts in Female Embryos

At the end of Müllerian development, the embryo displays dual gender phenotypic identity. Complete sex determination occurs afterward in two fundamental steps: (1) regression of the genetically unmatched reproductive tubes (Figure 1D) and (2) maintenance of the sex-matched ducts with their subsequent differentiation into adult reproductive organs (Figures 1E,F).

Throughout mammalian male sex differentiation, MIS (Müllerian-inhibiting substance) is secreted and binds to Misr2 (MIS type II receptor), activating the MIS signaling pathway that promotes Müllerian regression (Josso et al., 1993; Mishina et al., 1996; Kobayashi et al., 2004; Renfree et al., 2009). Misr2 is expressed in mouse Müllerian mesenchyme in both genders and is regulated by mesoepithelial-secreted Wnt7a (Baarends et al., 1994; Hacker et al., 1995; Parr and Mcmahon, 1998; Swain and Lovell-Badge, 1999; Kobayashi et al., 2004; Arango et al., 2008). Male mice bearing null mutations for MIS, *Misr2* or *Wnt7a* impair Müllerian duct regression (Behringer et al., 1994; Mishina et al., 1996; Parr and Mcmahon, 1998). A recent study investigating these *Misr2*^+^cells in female rodents has shown that they are kept in the postnatal uterine stroma where they are hypothesized to function as progenitors (Saatcioglu et al., 2019). This would differ from the human given that these cells disappear from the Müllerian mesenchyme by week 37 (Saatcioglu et al., 2019). In XX mouse embryos, Müllerian ducts persist in the absence of MIS whereas Wolffian ducts are cleared through the action of the transcription factor COUP-TFII (or NR2F2), expressed by the mouse Wolffian mesenchyme (Zhao et al., 2017) (Figure 1D).

After female sex determination, the Müllerian ducts are simple tubes comprised of an immature lumen and mesenchymal layers that starting a differentiation program. Post-development, a mesenchymal signature of *Hox* genes (mouse, human, chicken) and retinoic acid (RA) signaling (mouse) segmentally marking the ducts can be observed (Taylor et al., 1997; Ma et al., 1998; Nakajima et al., 2016, 2019; Ghosh et al., 2017) (Figure 1E). The *Hox/*RA signature consists of differentially expressed RA-related and *Hox* genes (*Hoxa9,10,11,13*) subluminally segmenting the proximal and distal Müllerian duct (Raines et al., 2013; Nakajima et al., 2016, 2019). Each specified Müllerian section gives rise to a different FRT organ (Figures 1E,F) with the most anterior differentiating into the oviducts.

The differential retinoic acid signaling consists of a decreasing gradient from the proximal (highest signal) to the caudal (signal inhibition) Müllerian mesenchyme where inhibition is mediated by the RA-degrading protein Cyp26a1 in mouse (Nakajima et al., 2016, 2019). Tuned activation and absence of the RA-signaling pathway determines oviduct-uterine and vaginal differentiation, respectively (Nakajima et al., 2016, 2019). In the former, RA signaling is active from E14.5 (post-Müllerian development) to P0 in mouse, when gradually decreases until disappearance by P15 (Nakajima et al., 2016). Hox genes are first detected at E15.5 in mouse, although *Hoxa10* has been documented in the middle duct at E14.5 (Taylor et al., 1997; Ma et al., 1998; Nakajima et al., 2016). At E16.5, *Hoxa9/10/11* have a similar expression pattern, marking the upper and middle segments of the Müllerian duct; however, *Hoxa9* extends further into half of the segment that will develop into oviducts. Conversely, *Hoxa13* is upregulated in the caudal region, which differentiate into the cervix and upper vagina. Within the first 2 weeks of mouse life the Hox genes restrict their expression to their determined organ; *Hoxa9* shows strong expression in the oviduct and low expression in the uterine horns, *Hoxa10* is robustly expressed in the uterine horns, *Hoxa11* is strongly expressed in the lower uterine horns and endocervix, and *Hoxa13* is moderately expressed in the uterine cervix and highly expressed in the ectocervix and upper vagina (Taylor et al., 1997; Ma et al., 1998) (Figure 1F). The *HoxD* cluster (*Hoxd9/10/11*) follows a similar fashion to their *Hoxa* paralogs (Dollé et al., 1991). This gene signature is conserved during adulthood in mouse, chicken and human. Additionally, Müllerian luminal differentiation has also been shown to be marked by mesoepithelial–epithelial transitions (MOETs). After female Müllerian development (where luminal cells retain a mesothelial phenotype), the epithelial marker *E*-cadherin is gradually expressed in female mouse luminal cells and they acquire epithelial apicobasal polarity (Orvis and Behringer, 2007; Stewart et al., 2013; Prunskaite-Hyyryläinen et al., 2016; Ghosh et al., 2017). These processes represent conserved MOETs observed in many species such as the human, mouse, chicken, quail, duck, goose, and rat (Dohr and Tarmann, 1984; Jacob et al., 1999). It is currently unknown whether this mechanism is derived from mesenchymal-specification signaling or is a cell-autonomous process that the Müllerian mesothelium undertakes.

Specific oviduct differentiation has been shown to rely on epithelial–mesenchymal interactions as demonstrated by mouse heterotypic recombinant assays at postnatal stages, where the epithelial identity of each Müllerian segment is determined by the associated mesenchyme (Yamanouchi et al., 2010). Mouse oviduct epithelium which has undertaken fate-decision endpoints has been detected at 3/4 weeks based on electron microscopy experiments and heterotypic recombinant assays (Komatsu and Fujita, 1978; Yamanouchi et al., 2010). Recombinant studies have also been performed with the other reproductive organs, leading to the same conclusions (Cunha, 1976; Kurita et al., 2001).

The proximal mouse Müllerian section with oviduct fate accounts for the highest RA activity (Nakajima et al., 2016), which suggests a role for this pathway in the differentiation mechanism. Cultured proximal Müllerian ducts at E14.5 differentiate into oviduct-like epithelial cells whereas middle Müllerian ducts differentiate into uterine-like epithelium (Nakajima et al., 2016). Inhibition of RA signaling through the pan-RAR antagonist AGN193109 in cultured anterior mouse Müllerian ducts induced differentiation of Trp63^+^vagina-like epithelia, and treatment with RA and AGN193109 rescued the oviduct epithelium phenotype and blocked the vagina-like induction (Nakajima et al., 2016). Taken together, it seems that a reversible fate decision has been made in the ducts at this embryonic point, although further research would be necessary to support this hypothesis. Regarding the specific role of Hox genes in oviduct patterning, no reproductive tract aberrations have been reported in mice lacking *Hoxa9/d9* (Fromental-Ramain et al., 1996; Raines et al., 2013). *Hoxa10*-null mice display anteriorization of the proximal first quarter of the uterine horns into oviducts, as well as homozygous *Hoxa11* knockouts (Satokata et al., 1995; Benson et al., 1996; Gendron et al., 1997). The expression of several genetic identities has been reported to influence oviduct differentiation. Reduction in *Wnt4* expression leads to aberrant mouse oviduct formation, whereas lack of *Wnt7a* prompts oviductal posteriorization with abnormal elongation and lack of coiling (Miller et al., 1998; Parr and Mcmahon, 1998; Vainio et al., 1999; Prunskaite-Hyyryläinen et al., 2016). *Ctnnb1* knocked-out Müllerian mesenchymal cells also trigger uncoiled oviducts, and its continuous stabilization prompts a failure in oviduct differentiation by impeding the expression of *E*-cadherin in the differentiating mesoepithelial Müllerian lumen (Deutscher and Hung-Chang Yao, 2007; Stewart et al., 2013). This suggests that fine tuning of the canonical WNT pathway is required for oviduct differentiation and that given the identical knockout phenotypes between Wnt7a knockouts and mesenchymal-specific *Ctnnb1* knockouts, Wnt7a could be a WNT effector candidate in this context. The cofactors Dach1/2 have also been proposed as contributors to oviductal differentiation given the hypoplastic oviduct in *Dach2^–/–^; Dach1^+/–^* females (Davis et al., 2008). Furthermore, an interesting event deserving additional investigation could be the differential epithelial *Lim1* expression that extendedly remains in the anterior mouse Müllerian region when it has disappeared from the rest of the duct (Kobayashi et al., 2004).

**FIGURE 4:**
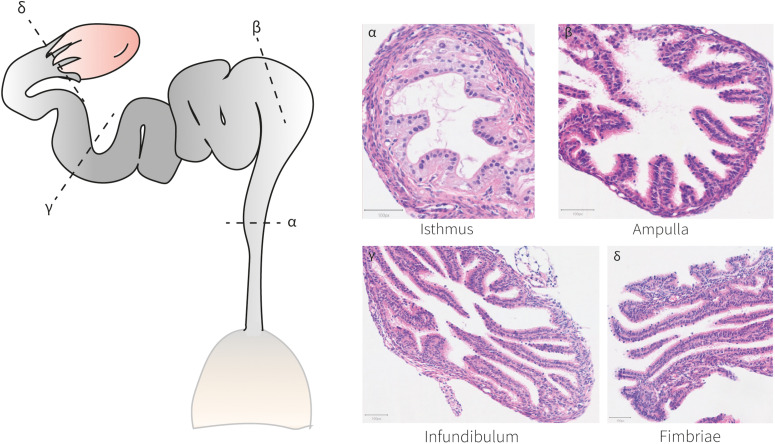
Histo-anatomical characterization of murine oviducts. Hematoxylin and eosin staining of oviduct sections shows the histo-anatomical pattern of the isthmus, ampulla, infundibulum and fimbriae following the posterior-anterior axis. The epithelium establishes luminal protrusions surrounded by a stromal and a smooth muscle layer.

At birth, mouse oviductal epithelial cells are distinguished by the expression of *Pax8* (Ghosh et al., 2017, 2020) (Figure 3). These *Pax8*^+^cells can self-renew and differentiate into *Pax8*^–^ ciliated cells by P3–P4 (Shi et al., 2014; Ghosh et al., 2017). After the first 2 weeks of life, the adult mouse oviducts become fully distinguishable and mature exhibiting four differentiated regions conserved across species; the isthmus, ampulla, infundibulum, and fimbriae (Figure 4). The mature human and mouse epithelium is composed of *Pax8*^+^secretory, *Pax8^–^ Caps*^+^ ciliated and intermediate secretory-ciliated cell subtypes (Hu et al., 2020). The Wnt non-canonical planar cell polarity (PCP) pathway has been reported to control diverse aspects of the mouse oviduct epithelial polarity and organ morphology through the action of Celsr1 from P2 to adulthood (Shi et al., 2014). In this study, the oviduct expression of other PCP pathway-related genes (*Celsr2*, *Vangl1/2*, *Dvl1/2*, *Pk2/3*, *Fz1-3/6/8* and *Ptk7*) was detected and further investigation on these entities could contribute to determine this pathways’ complete effector network. The role of Vangl2 controlling cell polarity in the mouse uterine horns has been previously shown, although the effects in the oviduct epithelial polarity has not been reported (Vandenberg and Sassoon, 2009). However, the authors did report a lack of oviduct coiling in mice with mutated *Vangl2*, which could be potentially linked to a collateral 50% reduction in *Wnt7a* expression (Vandenberg and Sassoon, 2009). Also, it would be valuable to study if this PCP pathway-dependent epithelial polarization develops at earlier stages, for example, at the differentiation time of Müllerian luminal MOETs.

### Genetics and Molecular Biology of Müllerian Duct Development and Differentiation

Several gene families regulate Müllerian duct development and oviduct differentiation. In this section, published genes with a role/phenotype in these processes are presented and discussed (Table 1). Additionally, our genome-wide survey of mouse knockouts identified novel genes potentially involved in Müllerian duct development and oviduct physiology and/or integrity (Supplementary Table 1).

**TABLE 1:** Gene expression and function in Müllerian duct and oviduct biology.

**Gene Family**	**Gene name**	**Symbol**	**Protein type**	**Expression**	**Function in Müllerian duct biology**
Paired box (Pax) [Group II]	Paired box gene 2	*Pax2*	Transcription factor (TF)	Coelomic mesoepithelial progenitors Müllerian mesoepithelial cells	Mesoepithelial progenitor specification Müllerian invagination
	Paired box gene 8	*Pax8*	TF	Coelomic mesoepithelial progenitors Müllerian mesoepithelial cells	Mesoepithelial progenitor specification Müllerian invagination
Wingless-type mouse mammary tumor virus (MMTV) integration site family (Wnt)	Wnt family member 4	*Wnt4*	Secreted signaling protein	Coelomic mesenchymal progenitors Mesonephric mesenchyme Müllerian mesenchymal cells	Müllerian invagination Müllerian elongation
	Wnt family member 7a	*Wnt7a*	Secreted signaling protein	Coelomic mesoepithelial progenitors Müllerian mesoepithelial cells	Upper Müllerian tract differentiation into oviducts and uterine horns
	Wnt family member 9b	*Wnt9b*	Secreted signaling protein	Wolffian epithelial cells	Müllerian duct elongation
Catenin	Catenin beta 1	*Ctnnb1*	Adhesion protein and transcriptional regulator	Stabilized in Müllerian mesenchymal cells	Stabilized protein: Hypoplastic oviduct Lack of epithelial differentiation in the mesoepithelial Müllerian duct before birth Knockout: Lack of oviduct coiling
Dach/dachshund	Dachshund family transcription factor 1 and 2	*Dach1* and *Dach2*	Transcriptional cofactors (COFs)	Müllerian mesoepithelial cells	Normal Müllerian duct development through control of Lim1 and Wnt7a expression and differentiation into oviducts
GATA	GATA binding protein 3	*Gata3*	TF	Wolffian epithelial cells	Müllerian duct elongation
DM (Doublesex/MAB-3) Domain	Doublesex and mab-3 related transcription factor 1	*Dmrt1*	TF	Coelomic mesenchymal progenitors Müllerian mesenchymal cells	Müllerian mesenchymal cell production and incorporation to the elongating Müllerian mesoepithelial tube
Retinoic acid receptors	Retinoic acid receptor alpha, retinoic acid receptor beta 2, retinoic acid receptor gamma	*RARα/RARβ2* and *RARα/RARγ*	Receptor	Müllerian duct (not specified)	Compound mutations in these genes impair Müllerian duct elongation
Nuclear Dbf2-related kinases	Large tumor suppressor kinase 1 and 2	*Lats1* and *Lats2*	Ser/Thr kinase	Müllerian mesenchymal cells	Maintenance of multipotency in Müllerian mesenchymal cells
Adhesion GPCR	Adhesion G-protein coupled receptor G1 or G protein-coupled receptor 56	*Adgrg1*	G protein-coupled receptor	Coelomic mesenchymal progenitors Müllerian mesenchymal cells	Müllerian duct elongation
TGF-β Type 1 receptor	Transforming Growth Factor Beta Receptor 1	*Tgfbr1*	TGF-β Type 1 receptor	Oviduct epithelium Oviduct mesenchyme and smooth muscle Müllerian Amhr2^+^ mesenchymal cells	Lack of this gene in Müllerian Amhr2^+^ mesenchymal cells results into cystic oviducts unable to carry out the reproductive function
Ribonuclease III (RNaseIII) family	Dicer 1, Ribonuclease III	*Dicer1*	RNaseIII	Ubiquitous	Lack of this gene in Müllerian Amhr2^+^ mesenchymal cells results into cystic oviducts with disorganized epithelium unable to carry out the reproductive function
LIM Homeobox (Lhx)	LIM homeobox 1	*Lim1/Lhx1*	TF	Coelomic mesoepithelial progenitors Müllerian mesoepithelial cells	Mesoepithelial progenitor specification Müllerian invagination
Homeobox	Empty spiracles homeobox 2	*Emx2*	TF	Coelomic mesoepithelial progenitors (?) Müllerian mesoepithelial cells	Müllerian duct development; unspecified affected stage
	HNF1 Homeobox B	*Hnf1b*	TF	Coelomic mesoepithelial progenitors	When absent, no Müllerian duct develops but no stage specified
	Homeobox a9, a10, a11, a13, d9, d10, d11, d13	*Hoxa9*, *Hoxa10*, *Hoxa11*, *Hoxa13*, *Hoxd9*, *Hoxd10*, and *Hoxd11*	TF	Müllerian mesenchymal cells Hoxa9/d9 in upper-middle region that becomes oviducts Hoxa10/d10 and Hoxa11/d11 in the middle region that becomes uterine horns Hoxa13 in the caudal region that becomes the cervix and upper vagina	Regional Müllerian epithelial differentiation In the adult FRT: Regulation of blastocyst implantation and initial development Hormone cycle

#### Pax2/8

Pax2/8 are master embryonic factors from the highly conserved Pax family, characterized by a paired box DNA-binding domain. Pax2/8 belong to the Pax2/5/8 (group II) subfamily which share a highly similar sequence containing a paired box domain, a partial homeodomain lacking a DNA-binding sequence and an octapeptide domain. Previous to coelomic mesoepithelial progenitor specification, *Pax2* is expressed in some mesonephric coelomic cells in mouse and chicken (Torres et al., 1995; Vainio et al., 1999; Pedersen et al., 2005; Guioli et al., 2007; Davis et al., 2008; Atsuta and Takahashi, 2016). In chickens, high levels of *Pax2* expression have been documented during the three developmental stages (Roly et al., 2020b). *Pax8* is expressed in the Müllerian duct from its initial development to later adult stages and mice bearing *Pax8* mutations present a normal Müllerian phenotype (Vainio et al., 1999; Bouchard et al., 2004; Carroll et al., 2005). In *Pax2*-null mice, Müllerian progenitor cells undergo initial invagination (Torres et al., 1995; Kobayashi et al., 2004) but do not elongate due to the absence of Wolffian ducts. In chickens, *Pax2* downregulation blocks Müllerian duct invagination (Atsuta and Takahashi, 2016). These opposite phenotypes are discussed later when data involving Pax8 and Lim1 are presented. In humans, the *PAX2* landscape is more complex. A *PAX2* polymorphism and a synonymous mutation have been linked to different Müllerian duct anomalies and a patient carrying a bicorporeal uterus and a double cervix, respectively (Wang et al., 2012; Xu et al., 2017). Furthermore, increased expression of this gene related to its hypomethylation was correlated to a septate uterus phenotype (Wang et al., 2020). This suggests that PAX2 might have a role in Müllerian development/differentiation in humans although this has not been conclusively proved as no mutations in this gene have been reported in Müllerian agenesis cases to date.

#### Lim1 and Hnf1b

Lim1 is a homeobox transcription factor with two amino-terminal cysteine-histidine-containing LIM domains that bind iron and zinc and a DNA-binding homeodomain (Barnes et al., 1994). In mice, its transcript levels peak in the embryo at E11.5 chronologically corresponding to Lim1-mediated coelomic progenitor specification (Barnes et al., 1994; Kobayashi et al., 2004). *Lim1* expression strongly persists during chicken and mouse Müllerian development (Kobayashi et al., 2004; Roly et al., 2020b). From E16.5–17.5, *Lim1* expression is restricted to the Müllerian anterior axis before complete disappearance (Kobayashi et al., 2004; Davis et al., 2008). In *Lim1*-null mouse and *Lim1*-downregulated chicken embryos, Müllerian invagination is blocked (Kobayashi et al., 2004; Atsuta and Takahashi, 2016). In humans, the same deletion in chromosome 17 encompassing *LIM1* causes Müllerian agenesis, also known as Mayer-Rokitansky-Küster-Hauser (MRKH) syndrome (Bernardini et al., 2009; Ledig et al., 2011; Nik-Zainal et al., 2011; Sandbacka et al., 2013; McGowan et al., 2015; Waschk et al., 2016). The phenotypes within MRKH patients are variable, for example, two different patients in the same study presented, the first, uterine and vaginal agenesis and the second, absence of the upper vagina with unicornuate uterus and bilaterally multicystic kidneys (Bernardini et al., 2009). This deletion is also the cause of a case of uterine fusion anomaly (Ledig et al., 2018). These are typical examples of mutations with incomplete penetrance and variable expressivity, which suggests that additional genetic and non-genetic factors control full penetrance in the human phenotypes. This deletion also includes the gene*HNF1β*, a POU homeodomain transcription factor expressed in the mesoepithelial progenitors during mouse Müllerian duct development as well as in the adult oviducts and uterine horns. Mice with homozygotic mutations in *Hnf1b* do not develop Müllerian ducts (Lokmane et al., 2010). Human mutations in *LIM1* have been associated with MRKH syndrome (Ledig et al., 2011, 2012; Sandbacka et al., 2013) whereas mutations in *HNF1β* have been related to fusion anomalies (Bingham et al., 2002).

In chicken embryos with shRNA-mediated *Pax2* knockdown, *Lim1* expression is dramatically decreased, hindering Müllerian duct invagination (Atsuta and Takahashi, 2016). In contrast, *Lim1* is still expressed in *Pax2*-null mouse Müllerian progenitor cells, and invagination occurs (Torres et al., 1995; Kobayashi et al., 2004). Additionally, *Lim1* mRNA levels are greatly reduced in the mesonephros of *Pax2^–/–^/Pax8^+/–^* mice (Boualia et al., 2013), suggesting that Pax2/8 might regulate *Lim1*. This potential regulation mechanism has not been elucidated but direct interaction between Lmx1b, another Lim-homeodomain family member, and Pax2 was proven by yeast two-hybrid assays and co-immunoprecipitation (Marini et al., 2005). The homeodomain was found to be essential for this event. Moreover, Pax2 directly binds *Lim1* enhancer regions in a Wolffian cell line model (Boualia et al., 2013). The apparent contradictory conclusions from the murine and chicken *Pax2*-deficient models, i.e., invagination/non-invagination outcomes upon Lim1 regulation, could be explained by Pax8 compensation. The mouse *Pax2* phenotype could be rescued by *Pax8* acting redundantly, whereas unlike in mammals, the chicken genome does not contain *Pax8*. This theory could be supported by two facts, (1) *Pax2* and *Pax8* are paralogous genes in the same subfamily and share the highest percentage of sequence similarity within Pax members, (2) Pax2/8 functional redundancy and synergistic cooperation with Lim1 was previously shown in renal system development in *Xenopus* and mice (Carroll and Vize, 1999; Bouchard et al., 2002).

#### Emx2

*Emx2* is a homeobox gene expressed in the mouse mesonephric coelomic epithelium at E10-11, although by E12-13 is restricted to the forming Müllerian mesoepithelium (Miyamoto et al., 1997; Pellegrini et al., 1997; Grote et al., 2008). In chicken, *Emx2* is highly expressed in the Müllerian ducts during their development (Roly et al., 2020b). *Emx2*-null mice completely lack Müllerian ducts (Miyamoto et al., 1997). In the Wolffian ducts, Pax2 is a direct transcriptional regulator of *Emx2* (Pedersen et al., 2005; Boualia et al., 2011), which could potentially be conserved in the Müllerian ducts. *Emx2* expression persists into mouse and human adulthood, is regulated by HoxA10 and is involved in the reproductive hormone cycle, blastocyst implantation and endometriosis (Du and Taylor, 2004, 2015).

#### Wnt4/Wnt7a/Wnt5a/Wnt9b/Ctnnb1

The WNT family of secreted glycoproteins is extensively involved in embryonic developmental processes, adult tissue differentiation and homeostasis. Concretely, Wnt4, Wnt5a, Wnt7a, and Wnt9b are WNT elicitors with an important role in Müllerian development and oviduct differentiation. In mouse and chicken, *Wnt4* is expressed in the Müllerian ridge before invagination and, subsequently, in the coelomic epithelium and Müllerian mesenchymal cells where the expression is maintained throughout fetal life (Vainio et al., 1999; Ayers et al., 2015; Prunskaite-Hyyryläinen et al., 2016). *Wnt4*-null mice show no Müllerian duct invagination and lack Müllerian structures (Vainio et al., 1999; Kobayashi et al., 2004; Orvis and Behringer, 2007; Prunskaite-Hyyryläinen et al., 2016), although *Wnt4* is also required during elongation (Prunskaite-Hyyryläinen et al., 2016). Conditional knockout of *Wnt4* and *Wnt5a* from mouse mesenchymal cells occasionally causes Müllerian aplasia as well (St-Jean et al., 2019a).

Mutations in *WNT4* have been found in patients with MRKH and hyperandrogenism (Biason-Lauber et al., 2004, 2007; Philibert et al., 2008, 2011). However, the individual role of this gene is not well delimited as *WNT4* mutations in MRKH patients not exhibiting hyperandrogenism could not be found (Ravel et al., 2009; Gervasini et al., 2010; Chang et al., 2012) and not all MRKH patients with hyperandrogenism have *WNT4* mutations.

*Wnt7a* is expressed in the murine Müllerian duct mesoepithelium throughout and after development, being restricted to the oviduct and uterine epithelium after birth (Miller et al., 1998; Vainio et al., 1999; Davis et al., 2008; Prunskaite-Hyyryläinen et al., 2016). In *Wnt7a*-null mice, the Müllerian duct develops, although the oviducts and uterine horns suffer posteriorization, where oviducts resemble uterine horns, and these acquire vaginal characteristics, promoting the postnatal loss of *Hoxa10/11* (Parr and Mcmahon, 1998; Miller et al., 1998). Oviductal regional differentiation occurs in *Wnt7a*-nulls, but coiling and normal elongation do not (Parr and Mcmahon, 1998). Its interaction with other Müllerian effectors is unknown but Wnt7a regulation of *Lim1* in the Müllerian duct has been discarded (Kobayashi et al., 2004). Furthermore, Wolffian mouse epithelium expresses *Wnt9b* during E9.5–14.5 to control Müllerian duct elongation by canonical WNT signaling (Carroll et al., 2005; Pedersen et al., 2005). In *Wnt9b* mutants, normal Müllerian development takes place until elongation arrest (Carroll et al., 2005). In Wolffian-specific *Lim1* conditional knockout embryos, *Cdh1* (*E*-cadherin) and *Wnt9b* expression is significantly reduced, triggering the same phenotype, and suggesting Wolffian Lim1-regulation of these genes (Pedersen et al., 2005). In humans, the importance of WNT9B in Müllerian formation and differentiation has proved inconclusive. On one hand, several *WNT9B* mutations in patients with Müllerian duct anomalies (Wang et al., 2014; Waschk et al., 2016) and a polymorphism linked to MRKH risk (Ma et al., 2015) have been reported. On the other hand, *WNT9B* was ruled out as a causative factor in 542 Chinese patients with Müllerian aberrations (Tang et al., 2014).

Furthermore, the expression of Wnt16, Wnt6, Wnt7b, Wnt2, and Wnt10a has been documented in the chicken developing Müllerian duct and they represent potential WNT candidates to be further explored (Roly et al., 2020b).

#### Retinoic Acid Receptors

Retinoic acid is a molecule derived from vitamin A that plays a crucial role in embryonic development (Mendelsohn et al., 1994). RA signaling is transduced by RARs and RXRs (retinoid X receptors), which are important during Müllerian duct development. Rarα^−/−/^Rarβ2^–/–^ mice block Müllerian development at E12.5 even though Wolffian ducts developed (Mendelsohn et al., 1994). The genotype Rarα^−/−/^Rarγ^–/–^ triggers the loss of the mouse Müllerian caudal end due to loss of the Wolffian caudal end (Mendelsohn et al., 1994). Furthermore, Rxrα^−/−/^Rarα^–/–^ lead to a loss of Müllerian ducts (Kastner et al., 1997). These observations suggest functional redundancy. Furthermore, the *Rara* and *Rarb* genes were found to be highly expressed in the developing Müllerian duct at constant levels along the three stages (Roly et al., 2020b). RA signaling has also been shown to play a role in Müllerian differentiation and the retinol dehydrogenase Rdh10 as well as the RA-synthesis enzyme Aldh1a2 regulated by the transcription factor C/ebpδ have been proposed to be the molecules acting in this pathway (Nakajima et al., 2016, 2019). More research to support these findings would be needed to unravel the complete RA signaling regulatory network. At post-birth stages, *Rara*, *Rarg*, *Rxra*, and *Rxrb* are expressed in the mouse uterus and vagina and it would be interesting to test whether this is conserved in the oviducts (Nakajima et al., 2016). Furthermore, some level of indirect Rar regulation on *Hoxa10* has been proposed, given that Rar does not bind to the *Hoxa10* promoter but RA-Rar signaling increased Hoxa10 expression (Nakajima et al., 2016).

#### Hox Genes

Hox genes are master embryonic regulators across animals. In vertebrates, they are organized into four unlinked genomic clusters, *HoxA/B/C/D*, located on chromosomes 6/11/15/2 in mouse; 7/17/12/2 in human; and 2/3/1/7 in chicken. They display temporal-spatial collinearity, meaning they are expressed in a regulated manner in time and space in the embryonic body according to their physical organization on the chromosome: genes nearer the 3′ end are expressed earlier and more anteriorly in the embryo than the next gene in the cluster. In adult mice, chickens, and humans, the *Hoxa9/10/11/13* and *Hoxd9/10/11/13* genes are expressed in the FRT mesenchyme following this spatial-specific pattern (*Hoxa9/d9* in oviducts, *Hoxa10/d10/a11/d11* in uterine horns and *Hoxa13/d13* cervix and vagina) (Taylor et al., 1997; Ma et al., 1998; Raines et al., 2013). These genes have been shown to control the FRT segmentation (Satokata et al., 1995; Benson et al., 1996; Gendron et al., 1997; Raines et al., 2013; Ashary et al., 2020). Functional redundancy has been suggested for the A and D clusters in the FRT, with stronger contribution from the HoxA cluster (Fromental-Ramain et al., 1996; Raines et al., 2013). Furthermore, redundancy and inter-regulation have been suggested among the *Hoxa10* and *Hoxa11* genes (Branford et al., 2000). *HoxA* genes are mainly expressed in the reproductive mesenchyme, although epithelial expression occurs during pregnancy initiation and the steroid hormone cycle (Benson et al., 1996; Ma et al., 1998). Hox genes are regulated by steroids through direct interaction with estrogen and progesterone receptors, which bind Hox regulatory elements (Gendron et al., 1997; Ma et al., 1998). Studies on *Hoxa10/11* genes reported a role not only during Müllerian duct differentiation but also blastocyst implantation and development, the hormone cycle, endometriosis and hydrosalpinx (Benson et al., 1996; Gendron et al., 1997; Du and Taylor, 2004, 2015; Xu et al., 2014; Ashary et al., 2020). Furthermore, a single-cell RNA sequencing study of Hox-mutant developing uterine horns has suggested a possible mechanism of action for these genes through the control of the Wnt and Cxcl12/Cxcr4 ligand/receptor pathways (Mucenski et al., 2019).

#### Other Genes

The Dach/dachshund genes are a conserved family of transcriptional cofactors. At E12.5–13.5, *Dach1/Dach2* are strongly expressed in the mouse Müllerian duct mesoepithelium (Davis et al., 2008). *Dach1* or *Dach2*-null mutants display a normal phenotype, but when both genes are mutated, Müllerian duct development is abnormal, suggesting functional redundancy as double heterozygotes appear normal (Davis et al., 2008). Dach1/2 regulate *Lim1* and *Wnt7a*, as their expression is nearly depleted in *Dach2^–/–^; Dach1^+/–^* and *Dach2^+/–^; Dach1^–/–^* females, and hypoplasia is evident in the FRT, with increasing severity along the anterior-posterior axis (Davis et al., 2008).

Dmrt1 is a transcription factor containing a zinc finger-like DNA-binding domain (DM domain) expressed in the chicken Müllerian coelomic placode. As duct development advances, it becomes expressed in Müllerian mesenchymal cells until ∼HH35 (Ayers et al., 2015). When *Dmrt1* is knocked down, Müllerian ridge thickening and mesenchymal cell production are compromised, often blocking elongation owing to the lack of mesenchymal cells (Ayers et al., 2015).

Lats1/2 (Large tumor suppressor kinase 1/2) are Ser/Thr kinases of the Hippo pathway acting as repressors of the transcriptional cofactors Yap and Taz (St-Jean et al., 2019b). The Hippo pathway, through the action of Lats1/2, is essential for maintaining Müllerian mesenchymal cell pluripotency. Conditional deletion of these genes results in increased aberrant expression of Yap, Taz and the Ctgf transcription factor, activating the myofibroblast differentiation program and causing aberrations in the Müllerian ducts and oviducts (St-Jean et al., 2019b). The gross defect in Müllerian ducts is an abnormal coiling during elongation which provokes the creation of multi-lumens in adult organs (St-Jean et al., 2019b). Defects in adult oviducts involved severe dilatation, caudal cysts that ruptured by adulthood breaking the oviduct in pieces (St-Jean et al., 2019b). However, a controlled expression of *Taz* and *Yap* might be necessary given that conditional inactivation of the Müllerian mesenchymal *Taz* and *Yap* triggers postnatal cystic aberrations on the oviduct smooth muscle layer impairing their reproductive function (Godin et al., 2020).

Gpr56 is an adhesion G protein-coupled receptor critical for chicken Müllerian elongation (Roly et al., 2020a). This protein is coded by the *Adgrg1* gene, is first expressed in chicken coelomic epithelium progenitors during invagination, and its expression is maintained in Müllerian luminal cells until later post-developmental stages (Roly et al., 2020a). When its expression is knocked down, the number of proliferative cells decreases *in vivo*, whereas its overexpression in a chicken fibroblastic cell line prompts an increase in cell proliferation and migration (Roly et al., 2020a), which suggest Gpr56 control on cell proliferation and migration during Müllerian duct elongation. Furthermore, this receptor family could play further or the same roles through different family members given that expression of G protein-coupled receptors is upregulated during Müllerian duct formation, with *Adgrd1* showing one of the highest expression levels at the last stages of development (Roly et al., 2020b).

Tgfbr1 is the type 1 receptor for TGF-β ligands controlling the transduction of the TGF-β signaling pathway. This pathway is transduced through intracellular SMAD proteins. In mouse oviducts, Tgfbr1 is expressed in the epithelium, mesenchymal and smooth muscle cells (Li et al., 2011). Conditional knockout of *Tgfbr1* in *Amhr2*^+^ mesenchymal Müllerian cells produces adult oviducts with cystic structures as a result of defects in smooth muscle development (Li et al., 2011). These structures prevented the reproductive function as seen by unsuccessful preimplantation embryo development and transport to the uterine horns. Furthermore, members of the TGF-β signaling pathway (Tgf-β1/2/3 and their corresponding receptors) have been identified in the developing chicken Müllerian duct and further investigation would reveal whether this pathway plays a role at early developmental stages as well (Roly et al., 2020b).

The gene *Dicer1* encodes an RNase III (Dicer) that processes microRNAs that modulate gene expression in the cytoplasm. In mice with *Amhr2*-Cre mediated conditional deletion of *Dicer1* (this RNase is missing in Müllerian mesenchymal cells), the oviducts present aberrations related to anomalies in the smooth muscle layer (Hong et al., 2008; Gonzalez and Behringer, 2009). In this model, the oviducts are shortened, cystic and the epithelium lacks organization. Furthermore, the isthmus presents a great loss of the smooth muscle layer and absence of epithelial folds. Successful reproduction is halted in these mice and most of the blastocysts were found near the ampulla, where fertilization happens, and never made it to establish a pregnancy in the uterine horns. Dicer regulates several microRNAs that are algorithm-predicted to regulate the expression of several genes related to Müllerian development and qPCR analysis demonstrated that the genes *Hoxa9*, *Hoxa10*, *Wnt5a*, *Wnt7a* were upregulated after *Dicer1* deletion in the Müllerian mesenchyme (Nagaraja et al., 2008).

#### Genome-Wide Screening of Gene Knockouts

**FIGURE 5:**
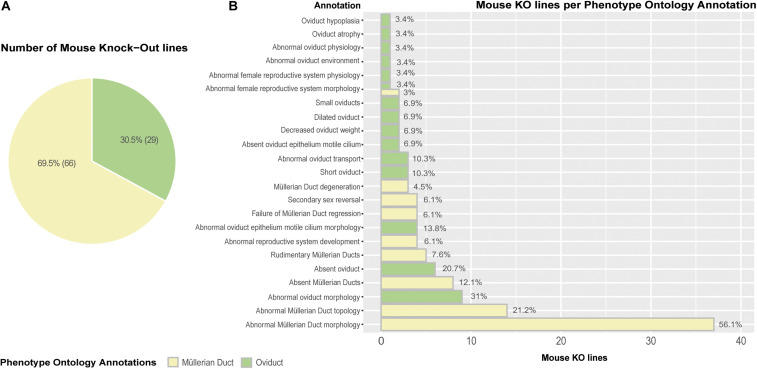
Mouse knock-out (KO) lines expressing Phenotype Ontology Annotations (POA) associated to Müllerian ducts/oviducts. **(A)** Pie chart representing KO lines in the MGI database registering at least one POA related to Müllerian duct/oviduct. **(B)** Barplot depicting the number of KO lines per annotation. Percentages are calculated as the number of mouse KO lines displaying a Phenotype Ontology Annotation out of the total number of mouse KO lines per POA type.

Lastly, we carried out an extensive genome-wide interrogation of mouse knockout lines reporting mutant phenotypes in Müllerian ducts/oviducts using the MGI (Mouse Genome Informatics) and DMDD (Deciphering the Mechanisms of Developmental Disorders) databases (Mohun et al., 2013; Smith et al., 2019). Ninety-five mouse knockout lines registered Phenotype Ontology Annotations (POA) affecting the Müllerian ducts (69.5%) and oviducts (30.5%) (Figure 5A). The mutant phenotypes with the highest incidence are abnormal Müllerian duct and oviduct morphology, abnormal Müllerian duct topology and absence of Müllerian ducts and oviducts (Figure 5B). Although the penetrance of most mutations was unknown, several genes previously unreported displayed significant penetrance, ranging from 33.3% for *Cir1* (Corepressor interacting with Rbpj, 1) and *Rpgrip1* (Rpgrip1-like) to 50% for *Actn4* (Alpha-actinin-4) and *Anks6* (Ankyrin repeat and sterile alpha motif domain containing 6), 72.7% for *Sh3pxd2a* (Sh3 and Px domains 2a) and 100% for *B9d2* (B9 domain containing 2), *Greb1l* (Greb1-like retinoic acid receptor coactivator), and *Nxn* (Nucleoredoxin) (Supplementary Table 1). Wolffian duct abnormalities might be behind the *Cir1*, *Anks6*, and *Greb1l* Müllerian phenotypes. We used the Database for Annotation, Visualization and Integrated Discovery (DAVID) v6.8 (Huang et al., 2009) for extracting the biological meaning of these genetic entities. The analysis showed that *Sh3pxd2a*, *B9d2*, *Rpgrip1*, and *Anks6* are cilium-related proteins involved in cell projection whereas *Cir1* is a repressor of the Notch signaling pathway. Although literature search of the possible relationship of these genes to Müllerian/oviduct biology was mostly unsuccessful, a link for *Greb1l* was found. In a *Greb1l* knock-out mouse, Müllerian ducts are missing (De Tomasi et al., 2017) and a study using whole-genome sequencing in patients with Müllerian agenesis found possible causative mutations in the *GREB1L* gene (Jacquinet et al., 2020). Greb1l has been proposed as a coactivator of RA-RAR mediated transcription in a study using zebrafish and mouse (Brophy et al., 2017) and a similar protein, GREB1, has been found to form a chromatin complex with estrogen receptor (ER) and RARs, among others, and to be indispensable for ER-mediated transcription in human cells (Mohammed et al., 2013).

In our search, knockout mice for widely reported *Pax2/8*, *Lhx1*, *Emx2*, *Hox* genes, *Wnt4/7a/9b*, and RARs, were also found. These mutant phenotypes correspond with literature reports, and they are among those genes registering mutant annotations of highest incidence and impact (Supplementary Table 1).

### Discussion

The Müllerian ducts form during embryonic development and anteriorly differentiates into the oviducts. In this review, a plausible model for Müllerian development and oviduct differentiation is proposed. In this model, *Pax2*^+^/*8*^+^/*Emx2*^+^/*Lim1*^+^ mesonephric coelomic epithelial cells undergo pEMT into Müllerian mesoepithelial cells, whereas *Dmrt1*^+^/*Wnt4*^+^ populations undergo EMT into Müllerian mesenchyme. The mesoepithelial progenitors give rise to the lumen through invagination controlled by cell intercalation-mediated apical contraction and subsequent rostral-caudal elongation in intimate contact with the Wolffian ducts. The BMP and FGF/RAS/MAPK signaling pathways have been shown to regulate, at least, early Müllerian developmental stages, but it would be necessary to establish their role in later developmental, differentiation and homeostatic stages. Elongation is regulated by several factors including mesonephric coelomic epithelium signaling, Wolffian-dependent canonical WNT pathway, PI3K/Akt pathway and Müllerian expressed genes. Signaling mediated by G protein-coupled receptors has been reported at these stages in chickens (Roly et al., 2020a, b). Furthermore, the TGF-β signaling pathway has been shown to play a role in Müllerian differentiation.

After Müllerian duct formation, mesoepithelial progenitors undergo MOETs, differentiating into true uncommitted epithelium before birth during a similar time window in which the mesenchymal Hox/RA signaling axis is established. RA signaling is required for oviduct and uterine differentiation and blocked for the establishment of vaginal epithelium. The primitive oviduct-committed epithelium further differentiates, as seen in the new-born mouse oviduct, where epithelial *Pax8*^+^ progenitors can self-renew and differentiate into ciliated and secretory epithelial cell subtypes. Committed oviduct cell types appear within the first weeks of life when full regional differentiation has been undertaken. The non-canonical Wnt PCP pathway has been established as the regulator of the epithelial polarization at these differentiation and adult stages. Interestingly, when smooth muscle differentiation is perturbed in models involving genes truncated in the Müllerian mesenchyme (e.g., *Lats1/2*, *Taz* and *Yap, Tgfbr1, Dicer*), there seems to be a common phenotype in the oviducts exhibiting fluid-filled cysts impeding the reproductive function.

A preliminary gene regulatory network (GRN) underlying Müllerian development and differentiation into oviducts has been described, and the components of this network have been hierarchically related. Furthermore, genome-wide survey of mouse knockout lines identified new genes involved in Müllerian and oviduct biology, which would benefit from further investigation. Some interesting questions to answer regarding the role of these new candidates in Müllerian/oviduct biology would be: (1) Do these genes interact with any of the genes in the Müllerian GRN? (2) What are their expression patterns and functions in Müllerian ducts development and oviduct differentiation and adult homeostasis? (3) Are there some gyneacological pathologies related to the identified genes in the female reproductive tract?

More research is also needed on the factors that trigger Müllerian placode formation and later developmental stages, molecular candidates in these networks, upstream regulators, downstream effector circuits, and the relationships among members. The current lack-of-function models and experiments could prove very informative for potential future studies. Furthermore, there is limited research on prenatal Müllerian differentiation into oviducts. In more studied reproductive organs such as the vagina, important investigations of the mesenchymal-epithelial interactions during Müllerian differentiation have allowed the discovery of the GRN underlying this process. In the vagina, the Bmp4/Smad, ActA/Runx1, and Fgf7/10/Fgfr2IIIb/Mapk pathways independently specify Müllerian differentiation into the vaginal epithelium (Laronda et al., 2013; Terakawa et al., 2016, 2020). This is an elegant example of mesenchymal-epithelial interactions where the mesenchymal-secreted factors *BMP4*, *FGF7/10* and Activin A specify the immature caudal Müllerian epithelium into vaginal epithelium. Studies like this in the oviducts would greatly advance our understanding of oviduct-specific Müllerian differentiation. Investigation on postnatal oviduct morphogenesis and genetic programs, cell identities and signaling pathways underlying these changes are scarce and would represent an exciting new direction on the field. Additionally, information on the differentiation/maintenance programs executed by adult oviduct epithelial cells is also lacking. Finally, taken together, this review could serve as a guide to future research choices aimed at improving our understanding of Müllerian developmental processes, oviduct differentiation, FRT biology and their associated pathologies.

## Author Contributions

LS: conceptualization, methodology, investigation, and writing – original draft. IR, MM, MA, ZH, NW, AbA, AsA, TS-S, and AAA: writing – review and editing. LS and IR: visualization. AAA: supervision. All authors contributed to the article and approved the submitted version.

## Conflict of Interest

The authors declare that the research was conducted in the absence of any commercial or financial relationships that could be construed as a potential conflict of interest.
