# Supplementary material for: Mechanistic Drivers of Müllerian Duct Development and Differentiation Into the Oviduct
Source: Front Cell Dev Biol. 2021 Mar 8;9:605301. doi: 10.3389/fcell.2021.605301 (PMC7982813; doi:10.3389/fcell.2021.605301)
Supplement: Supplementary file 1 [file Data_Sheet_1.pdf]

Allelic Composition	Genetic background	Gene Symbol	Gene name	Mutation penetrance	Annotated Term	Reference	Mutant phenotype	Further mutant phenotypical information	Database
1700067K01Rik$^{tm2a(KOMP)Wtsi}$/1700067K01Rik$^{tm2a(KOMP)Wtsi}$	(C57BL/6N-1700067K01Rik$^{tm2a(KOMP)Wtsi}$/Wtsi)	1700067K01Rik	RIKEN cDNA 1700067K01 gene	2 out of 8	abnormal Mullerian duct morphology	J:239583	Müllerian duct	Right side distant segment absent; Left side distant segment absent	MGI, DMDD
4933434E20Rik$^{tm1a(EUCOMM)Wtsi}$/4933434E20Rik$^{tm1a(EUCOMM)Wtsi}$	(C57BL/6N-4933434E20Rik$^{tm1a(EUCOMM)Wtsi}$/Wtsi)	4933434E20Rik	RIKEN cDNA 4933434E20 gene	1 out of 6	abnormal Mullerian duct morphology	J:239583	Müllerian duct	Right distal segment forms a hook	MGI, DMDD
Actn4$^{tm1a(EUCOMM)Wtsi}$/Actn4$^{tm1a(EUCOMM)Wtsi}$	(C57BL/6N-Actn4$^{tm1a(EUCOMM)Wtsi}$/Wtsi)	Actn4	Actinin alpha 4	3 out of 6	abnormal Mullerian duct morphology	J:239583	Müllerian duct	Left, distal segment missing; Right, distal segment missing;	MGI, DMDD
Adamts3$^{tm1b(KOMP)Wtsi}$/Adamts3$^{tm1b(KOMP)Wtsi}$	(C57BL/6N-Adamts3$^{tm1b(KOMP)Wtsi}$/Wtsi)	Adamts3	A disintegrin-like and metallopeptidase (reprolysin type) with thrombospondin type 1 motif, 3	1 out of 7	abnormal Mullerian duct morphology	J:239583	Müllerian duct	Left distal segment forms a hook. At the same time, left Wolffian duct has a cystic enlargement at connection to bladder	MGI, DMDD
		Adamts3	A disintegrin-like and metallopeptidase (reprolysin type) with thrombospondin type 1 motif, 3	1 out of 7	abnormal Mullerian duct topology	J:239583	Müllerian duct	Right distal segment forms a hook	MGI, DMDD
Adcy9$^{tm1b(EUCOMM)Wtsi}$/Adcy9$^{tm1b(EUCOMM)Wtsi}$	(C57BL/6N-Adcy9$^{tm1b(EUCOMM)Wtsi}$/Wtsi)	Adcy9	Adenylate cyclase 9	1 out of 8	abnormal Mullerian duct morphology	J:239583	Müllerian duct	Missing left distal segment	MGI, DMDD
		Adcy9	Adenylate cyclase 9	1 out of 8	abnormal Mullerian duct topology	J:239583	Müllerian duct	Right distal segment forms a hook	MGI, DMDD
Amhr2^{tm1Bhr}/Amhr2^{tm1Bhr}	(involves: 129S7/SvEvBrd)	Amhr2	Anti-Mullerian hormone type 2 receptor	No information	failure of Mullerian duct regression	J:171430	Müllerian duct	Normal Müllerian ducts in female, but failure of Müllerian duct regression in males	MGI
Amhr2$^{tm3(cre)Bhr}$/Amhr2+ ; Ctnnb1^{tm2Kem}/Ctnnb1$^{tm2.1Kem}$	(involves: 129S1/Sv * 129S7/SvEvBrd * 129X1/SvJ)	Amhr2; Ctnnb1	Anti-Mullerian hormone type 2 receptor; Catenin (cadherin associated protein), beta 1	No information	abnormal Mullerian duct morphology	J:171430	Müllerian duct	Mesenchyme is less condensed	MGI, DMDD
		Amhr2; Ctnnb1	Anti-Mullerian hormone type 2 receptor; Catenin (cadherin associated protein), beta 1	No information	failure of Mullerian duct regression	J:171430	Müllerian duct	Wild-type Müllerian ducts in female not reported, but failure of Müllerian duct regression in males	MGI, DMDD
Anks6$^{tm1b(KOMP)Wtsi}$/Anks6$^{tm1b(KOMP)Wtsi}$	(C57BL/6N-Anks6$^{tm1b(KOMP)Wtsi}$/Wtsi)	Anks6	Ankyrin repeat and sterile alpha motif domain containing 6	1 out of 2	abnormal Mullerian duct morphology	J:239583	Müllerian duct	Left, distal segment ends blind. Both Wolffian ducts have dstal ends blind as well in all the mutants (penetrance 2/2)	MGI, DMDD
Ar^{Tfm}/Y	involves: NMRI * STOCK EdaTa Atp7aMo-blo	Ar	Androgen receptor	No information	secondary sex reversal	J:140803	Müllerian duct	The Müllerian ducts fail to regress	MGI, DMDD
	involves: STOCK EdaTa Atp7aMo-blo	Ar	Androgen receptor	No information	secondary sex reversal	J:140803	Müllerian duct	The Müllerian ducts fail to regress	MGI, DMDD
	STOCK EdaTa Atp7aMo-blo/+ +	Ar	Androgen receptor	No information	secondary sex reversal	J:140803	Müllerian duct	The Müllerian ducts fail to regress	MGI, DMDD
		Ar	Androgen receptor	No information	abnormal female reproductive system morphology	J:5173	Müllerian duct	The Müllerian ducts fail to regress	MGI, DMDD
Atp11a$^{tm1a(KOMP)Wtsi}$/Atp11a$^{tm1a(KOMP)Wtsi}$	(C57BL/6N-Atp11a$^{tm1a(KOMP)Wtsi}$/Wtsi)	Atp11a	ATPase, class VI, type 11A	2 out of 5	abnormal Mullerian duct morphology	J:239583	Müllerian duct	Left side, most distal segment missing	MGI, DMDD
Auts2$^{em1(IMPC)Wtsi}$/Auts2$^{em1(IMPC)Wtsi}$	(C57BL/6N-Auts2$^{em1(IMPC)Wtsi}$/Wtsi)	Auts2	Autism susceptibility candidate 2	1 out of 4	abnormal Mullerian duct topology	J:239583	Müllerian duct	Left distal segment forms a hook	MGI, DMDD
B9d2$^{tm1a(EUCOMM)Wtsi}$/B9d2$^{tm1a(EUCOMM)Wtsi}$	(C57BL/6N-B9d2$^{tm1a(EUCOMM)Wtsi}$/Wtsi)	B9d2	B9 protein domain 2	4 out of 4	abnormal Mullerian duct morphology	J:239583	Müllerian duct	Right and left distal segment missing, or left side, distal segment missing	MGI, DMDD
Brd2$^{em1(IMPC)Wtsi}$/Brd2$^{em1(IMPC)Wtsi}$	(C57BL/6N-Brd2$^{em1(IMPC)Wtsi}$/WtsiIeg)	Brd2	Bromodomain containing 2	1 out of 5	abnormal Mullerian duct topology	J:239583	Müllerian duct	Right distal segment forms a hook	MGI, DMDD
Cbx6$^{tm1a(EUCOMM)Wtsi}$/Cbx6$^{tm1a(EUCOMM)Wtsi}$	(C57BL/6N-Cbx6$^{tm1a(EUCOMM)Wtsi}$/Wtsi)	Cbx6	Chromobox 6	1 out of 7	abnormal Mullerian duct morphology	J:239583	Müllerian duct	Left side, most distal segment missing	MGI, DMDD
		Cbx6	Chromobox 6	1 out of 7	abnormal Mullerian duct topology	J:239583	Müllerian duct	Left distal segment forms a hook	MGI, DMDD
Cfap53$^{em1(IMPC)Wtsi}$/Cfap53$^{em1(IMPC)Wtsi}$	(C57BL/6N-Cfap53$^{em1(IMPC)Wtsi}$/Wtsi)	Cfap53	Cilia and flagella associated protein 53	1 out of 5	abnormal Mullerian duct morphology	J:239583	Müllerian duct	Right distal segment missing	MGI, DMDD
Chst11$^{tm1a(KOMP)Wtsi}$/Chst11$^{tm1a(KOMP)Wtsi}$	(C57BL/6N-Chst11$^{tm1a(KOMP)Wtsi}$/Wtsi)	Chst11	Carbohydrate sulfotransferase 11	1 out of 10	abnormal Mullerian duct morphology	J:239583	Müllerian duct	Right distal segment missing	MGI, DMDD
Cir1$^{tm3a(KOMP)Wtsi}$/Cir1$^{tm3a(KOMP)Wtsi}$	(C57BL/6N-Cir1$^{tm3a(KOMP)Wtsi}$/Wtsi)	Cir1	Corepressor interacting with RBPJ, 1	1 out of 3	abnormal Mullerian duct morphology	J:239583	Müllerian duct	Right and left distal segment missing. Due to Wolffian duct, which joins sac-like extension of bladder	MGI, DMDD
Cmip$^{tm1a(EUCOMM)Wtsi}$/Cmip$^{tm1a(EUCOMM)Wtsi}$	(C57BL/6N-Cmip$^{tm1a(EUCOMM)Wtsi}$/Wtsi)	Cmip	C-Maf inducing protein	1 out of 10	abnormal Mullerian duct morphology	J:239583	Müllerian duct	Right distal Müllerian duct malformed	MGI, DMDD
Cpt2$^{tm1b(KOMP)Wtsi}$/Cpt2$^{tm1b(KOMP)Wtsi}$	(C57BL/6N-Cpt2$^{tm1b(KOMP)Wtsi}$/Wtsi)	Cpt2	Carnitine palmitoyltransferase 2	1 out of 6	abnormal Mullerian duct morphology	J:239583	Müllerian duct	Left distal segment missing	MGI, DMDD
		Cpt2	Carnitine palmitoyltransferase 2	1 out of 6	abnormal Mullerian duct topology	J:239583	Müllerian duct	Distal segment forms a hook	MGI, DMDD
Crim1$^{em1(IMPC)Wtsi}$/Crim1$^{em1(IMPC)Wtsi}$	(C57BL/6N-Crim1$^{em1(IMPC)Wtsi}$/WtsiOrl)	Crim1	Cysteine rich transmembrane BMP regulator 1 (chordin like)	2 out of 10	abnormal Mullerian duct topology	J:239583	Müllerian duct	Distal segment forms a hook	MGI, DMDD
Cyp26a1$^{tm1.1Ptk}$/Cyp26a1$^{tm1.1Ptk}$		Cyp26a1	Cytochrome P450, family 26, subfamily a	No information	abnormal reproductive system development	J:67399	Müllerian duct	Mesonephric and paramesonephric ducts merged prematurely within a hypoplastic urogenital sinus. Both ducts are blind at their end.	MGI, DMDD
Dlg1^{tm1Taki}/Dlg1^{tm1Taki}	(involves: C57BL/6 * CBA)	Dlg1	Discs large MAGUK scaffold protein 1	No information	abnormal Mullerian duct morphology	J:121262	Müllerian duct	At E13.5 the lower end of the Mullerian duct has not elongated to the bladder wall, at E14.5, the Mullerian duct remains connected to the ureter and in some cases the common nephric duct, at E15.5 and E18.5, the Mullerian duct has not fused laterally, are constricted and the lower end does not separate from the ureter. A abnormal Wolffian duct morphology (J:121262) is also involved. At E14.5 and E18.5, ureters retain a connection to the Wolffian duct.	MGI, DMDD
Ehbp1l1$^{tm1a(EUCOMM)Wtsi}$/Ehbp1l1$^{tm1a(EUCOMM)Wtsi}$	(C57BL/6N-Ehbp1l1$^{tm1a(EUCOMM)Wtsi}$/Wtsi)	Ehbp1l1	EH domain binding protein 1-like 1	1 out of 6	abnormal Mullerian duct morphology	J:239583	Müllerian duct	Right distal segment missing	MGI, DMDD
		Ehbp1l1	EH domain binding protein 1-like 1	2 out of 6	abnormal Mullerian duct topology	J:239583	Müllerian duct	Left or right distal segment forms a hook	MGI, DMDD
Emx2^{tm1Sia}/Emx2^{tm1Sia}	(involves: C57BL/6 * CBA)	Emx2	Empty spiracles homeobox 2	No information	absent Mullerian ducts	J:40605	Müllerian duct	Mullerian ducts are absent at E13 and Wolffian ducts degenerate at E11.5	MGI, DMDD
Fit5^{1R}/Fit5^{1R}	R:D7R75M	Fit5	Fitness 5	No information	abnormal reproductive system development	J:100221	Müllerian duct	Entire reproductive tract is underdeveloped and small	MGI
Flvcr2$^{tm1a(EUCOMM)Hmgu}$/Flvcr2$^{tm1a(EUCOMM)Hmgu}$	(C57BL/6N-Flvcr2$^{tm1a(EUCOMM)Hmgu}$/Wtsi)	Flvcr2	Feline leukemia virus subgroup C cellular receptor 2	2 out of 5	abnormal Mullerian duct topology	J:239583	Müllerian duct	Right duct conected to Wolffian duct before entering the bladder, another embryo left distal segment forms a hook and the left Wolffian duct on the same embryo has an abnormal connection with ureter	MGI, DMDD
Gas2l2$^{tm1a(KOMP)Wtsi}$/Gas2l2$^{tm1a(KOMP)Wtsi}$	(C57BL/6N-Gas2l2$^{tm1a(KOMP)Wtsi}$/Wtsi)	Gas2l2	Growth arrest-specific 2 like 2	1 out of 8	abnormal Mullerian duct topology	J:239583	Müllerian duct	Right duct ends blind	MGI, DMDD
Ggt1^{tm1Zuk}/Ggt1^{tm1Zuk}	involves: 129S7/SvEvBrd * C57BL/6J	Ggt1	Gamma-glutamyltransferase 1	No information	abnormal reproductive system development	J:34309	Oviducts	Reproductive tracts of both females and males are hypoplastic	MGI
Greb1l^{em1Cjea}/Greb1l^{em1Cjea}	(involves: C57BL/6J)	Greb1l	Growth regulation by estrogen in breast cancer-like	100%	absent Mullerian ducts	J:248902	Müllerian duct	At E13.5, all embryos show absence of Mullerian ducts, at E13.5, all embryos show absence of Wolffian ducts	MGI, DMDD
Gt(ROSA)26Sor$^{tm2(Wnt1/GFP)Amc}$/Gt(ROSA)26Sor+ ; Wnt9b$^{tm1.1Amc}$/Wnt9b$^{tm1.1Amc}$	(involves: 129X1/SvJ * C57BL/6 * CBA)	Wnt9b	Wingless-type MMTV integration site family, member 9B	100%	abnormal Mullerian duct morphology	J:100575	Müllerian duct	Müllerian development is arrested	MGI, DMDD
H13$^{tm1b(KOMP)Wtsi}$/H13$^{tm1b(KOMP)Wtsi}$	(C57BL/6N-H13$^{tm1b(KOMP)Wtsi}$/Wtsi)	H13	Histocompatibility 13	6 out of 11	abnormal Mullerian duct morphology	J:239583	Müllerian duct	Left distal segment missing (x3 embryos), left missing and right distal segment missing, ends with a bulb (x1 embryo), Right and left distal segment missing (x1 embryo), right and left distal segment missing and right forms hook (x1 embryo)	MGI, DMDD
		H13	Histocompatibility 13	1 out of 11	absent Mullerian ducts	J:239583	Müllerian duct	Left duct missing	MGI, DMDD
Insl3^{tm1Imad}/Insl3^{tm1Imad}	involves: 129/Sv * CD-1	Insl3	Insulin-like 3	No information	abnormal reproductive system development	J:83429	Müllerian duct	Mullerian duct derivatives were absent	MGI, DMDD
Itga3^{tm1Jak}/Itga3^{tm1Jak} ; Itga6^{tm1Egl}/Itga6^{tm1Egl}	(involves: 129S2/SvPas * 129S4/SvJae)	Itga3, Itga6	Integrin alpha 3, Integrin alpha 6	No information	abnormal Mullerian duct morphology	J:56564	Müllerian duct	Caudal portions of the Mullerian ducts are absent or interrupted. Wolffian ducts exhibit the same phenotype.	MGI, DMDD
Kifbp$^{tm1a(KOMP)Wtsi}$/Kifbp$^{tm1a(KOMP)Wtsi}$	(C57BL/6N-Kifbp$^{tm1a(KOMP)Wtsi}$/Wtsi)	Kifbp	Kinesin family binding protein	No information	abnormal Mullerian duct morphology	J:239583	Müllerian duct	No information	MGI, DMDD
Lamc1^{tm1Umr}/Lamc1^{tm1Umr}	(either: (involves: 129S1/Sv * 129X1/SvJ) or (involves: 129S1/Sv * 129X1/SvJ * C57BL/6))	Lamc1	Laminin, gamma 1	No information	abnormal Mullerian duct morphology	J:76368	Müllerian duct	At E13.5, the Mullerian duct is blind-ending and abnormal Wolffian duct morphology is present as in 18 embryos lacking metanephric kidney induction, the Wolffian duct is blind-ending and at E10.5, the basement membranes of the elongating Wolffian duct is discontinuous whereas it is continuous in wild-type mice	MGI, DMDD
Lhfpl2^{vgim}/Lhfpl2^{vgim}	(B6(129S4)-Lhfpl2^{vgim}/GrsrJ)	Lhfpl2	Lipoma HMGIC fusion partner-like 2	No information	abnormal Mullerian duct topology	J:241136	Müllerian duct	Homozygotes at E15.5 have Mullerian ducts with enlarged tips that fail to merge or enter the urogenital sinus, and this results in unmerged upper vagina and lower vaginal agenesis. Abnormal Wolffian duct connection (J:241136) at E15.5 the tips of the Wolffian ducts are enlarged and flattened and fail to enter the urogenital sinus, which results in the convolutions at the junctions of vas deferens and urethra	MGI, DMDD
Lhx1^{tm1Tmj}/Lhx1$^{tm2.1Bhr}$; Tg(Pax2-cre)10Shwl/0	(involves: 129S1/SvEv * C57BL/6 * CD-1 * SJL)	Lhx1	LIM homeobox protein 1	No information	rudimentary Mullerian ducts	J:104938	Müllerian duct	Only rostral-most part of duct forms in mutants and at E10.75, extension of the nephric duct to urogenital sinus is impaired	MGI, DMDD
Lhx1$^{tm2.1Bhr}$/Lhx1^{tm1Tmj} ; Tg(Hoxb7-cre)13Amc/0	(involves: 129S/SvEv * C57BL/6)	Lhx1	LIM homeobox protein 1	No information	abnormal Mullerian duct morphology	J:98831	Müllerian duct	Only rostral-most part of duct forms in mutants and at E10.75, extension of the nephric duct to urogenital sinus is impaired, Mullerian duct degeneration adjacent to where the Wolffian duct was lost	MGI, DMDD
		Lhx1	LIM homeobox protein 1	No information	absent Mullerian ducts	J:98831	Müllerian duct	Only rostral-most part of duct forms in mutants and at E10.75, extension of the nephric duct to urogenital sinus is impaired, Mullerian duct degeneration adjacent to where the Wolffian duct was lost	MGI, DMDD
		Lhx1	LIM homeobox protein 1	No information	Mullerian duct degeneration	J:98831	Müllerian duct	Only rostral-most part of duct forms in mutants and at E10.75, extension of the nephric duct to urogenital sinus is impaired, Mullerian duct degeneration adjacent to where the Wolffian duct was lost	MGI, DMDD
		Lhx1	LIM homeobox protein 1	No information	rudimentary Mullerian ducts	J:98831	Müllerian duct	Only rostral-most part of duct forms in mutants and at E10.75, extension of the nephric duct to urogenital sinus is impaired, Mullerian duct degeneration adjacent to where the Wolffian duct was lost	MGI, DMDD
Lhx1^{tm1Bhr}/Lhx1^{tm1Tmj}	involves: 129S/SvEv * C57BL/6	Lhx1	LIM homeobox protein 1	100%	abnormal female reproductive system morphology	J:98831	Müllerian duct	Females lacked the reproductive tract but had normal ovaries	
Lhx9^{tm1Lmgd}/Lhx9^{tm1Lmgd}	involves: 129S1/Sv * 129X1/SvJ * C57BL/6	Lhx9	LIM homeobox protein 9	No information	secondary sex reversal	J:60621	Müllerian duct	All mice phenotypically female, although half were genetically male. In these males, no male accessory sex organs found.	MGI, DMDD
Morc2a$^{em1(IMPC)Wtsi}$/Morc2a$^{em1(IMPC)Wtsi}$	(C57BL/6N-Morc2a$^{em1(IMPC)Wtsi}$/WtsiOulu)	Morc2a	Microrchidia 2A	3 out of 11	absent Mullerian ducts	J:239583	Müllerian duct	Ducts absent in both sides. Distal segment missing in both sides.	MGI, DMDD
Myo9a$^{tm1b(EUCOMM)Wtsi}$/Myo9a$^{tm1b(EUCOMM)Wtsi}$	(C57BL/6N-Myo9a$^{tm1b(EUCOMM)Wtsi}$/Wtsi)	Myo9a	Myosin IXa	1 out of 6	abnormal Mullerian duct morphology	J:239583	Müllerian duct	Left distal segment forms a hook	MGI, DMDD
Nxn$^{tm1b(EUCOMM)Wtsi}$/Nxn$^{tm1b(EUCOMM)Wtsi}$	(C57BL/6N-Nxn$^{tm1b(EUCOMM)Wtsi}$/Wtsi)	Nxn	Nucleoredoxin; targeted mutation 1b	3 out of 3	abnormal Mullerian duct morphology	J:239583	Müllerian duct	Left distal segment forms a hook, righ distal segment splits , most of left Müllerian duct segment absent	MGI, DMDD
Pax2^{tm1Mbu}/Pax2^{tm1Mbu}	(C3.129P2-Pax2^{tm1Mbu})	Pax2	Paired box 2	No information	Mullerian duct degeneration	J:63683	Müllerian duct	Mullerian ducts initially form in the anterior region of the genital ridge but subsequently degenerate due to Wolffian degeneration	MGI, DMDD
Pax2^{tm1Pgr}/Pax2^{tm1Pgr}	(involves: 129S1/Sv * 129X1/SvJ)	Pax2	Paired box 2	No information	Mullerian duct degeneration	J:30343	Müllerian duct	At E16.5, the parts of Mullerian ducts initially formed have degenerated, given that the Wolffian ducts have degenerated, and are contained within the remnant of urogenital ridges flanking the gonads	MGI, DMDD
		Pax2	Paired box 2	No information	rudimentary Mullerian ducts	J:30343	Müllerian duct	At E13.5, mutant Mullerian ducts are only present at the uppermost parts of the genital ridge, whereas wild-type ducts are found along the entire length of the genital ridge and have reached the cloaca	MGI, DMDD
Pax8^{tm1Pgr}/Pax8^{tm1Pgr}	(either: (involves: 129S1/Sv * 129X1/SvJ) or (involves: 129S1/Sv * 129X1/SvJ * C57BL/6))	Pax8	Paired box 8	No information	abnormal Mullerian duct morphology	J:129545	Müllerian duct	T4-substituted female homozygotes show abnormal postnatal differentiation of the Mullerian duct	MGI, DMDD
Pdzk1$^{tm2b(EUCOMM)Wtsi}$/Pdzk1$^{tm2b(EUCOMM)Wtsi}$	(C57BL/6N-Pdzk1$^{tm2b(EUCOMM)Wtsi}$/Wtsi)	Pdzk1	PDZ domain containing 1	2 out of 9	abnormal Mullerian duct morphology	J:239583	Müllerian duct	Left, distal segment missing; Right, distal segment missing	MGI, DMDD
Rala$^{tm1a(EUCOMM)Wtsi}$/Rala$^{tm1a(EUCOMM)Wtsi}$	(C57BL/6N-Rala$^{tm1a(EUCOMM)Wtsi}$/Wtsi)	Rala	V-ral simian leukemia viral oncogene A (ras related)	No information	abnormal Mullerian duct morphology	J:239583	Müllerian duct	Right distal segment missing, also right Wolffian duct's morphology is abnormal	MGI, DMDD
Rara^{tm1Ipc}/Rara^{tm1Ipc} ; Rarb^{tm1Ipc}/Rarb^{tm1Ipc}	(involves: 129S2/SvPas)	Rara, Rarb	Retinoic acid receptor, alpha, Retinoic acid receptor, beta	100%	absent Mullerian ducts	J:21034	Müllerian duct	The paramesonephric ducts are absent despite the presence of normal Wolffian ducts resulting in the loss of the uterine tubes, uterus and the cranial vagina which are all derived from the paramesonephric ducts	MGI, DMDD
Rara^{tm1Ipc}/Rara^{tm1Ipc} ; Rarb^{tm1Mma}/Rarb^{tm1Mma}	(involves: 129S2/SvPas * C57BL/6)	Rara, Rarb	Retinoic acid receptor, alpha, Retinoic acid receptor, beta	100%	absent Mullerian ducts	J:43344	Müllerian duct	Agenesis of the Mullerian duct	MGI, DMDD

Rara^{tm1Ipc}/Rara^{tm1Ipc} ; Rarg^{tm1Ipc}/Rarg^{tm1Ipc}	(involves: 129S2/SvPas)	Rara, Rarg	Retinoic acid receptor, alpha, Retinoic acid receptor, gamma	No information	rudimentary Mullerian ducts	J:21034	Müllerian duct	The caudal paramesonephric ducts are absent in females resulting in the loss of the body of the uterus and the cranial vagina, at E18.5 which are derived from these ducts. This is due to the caudal absence in Wolffian ducts	MGI, DMDD
Rara^{tm2Ipc}/Rara^{tm2Ipc} ; Rarb^{tm1Ipc}/Rarb^{tm1Ipc}	(involves: 129S2/SvPas)	Rara, Rarb	Retinoic acid receptor, alpha, Retinoic acid receptor, beta	100%	rudimentary Mullerian ducts	J:21034	Müllerian duct	Caudal paramesonephric ducts are absent in females at E13.5	MGI, DMDD
Rpgrip1l$^{tm1a(EUCOMM)Wtsi}$/Rpgrip1l$^{tm1a(EUCOMM)Wtsi}$	(C57BL/6N-Rpgrip1l$^{tm1a(EUCOMM)Wtsi}$/Wtsi)	Rpgrip1l	Rpgrip1-like	1 out of 3	abnormal Mullerian duct topology	J:239583	Müllerian duct	Right distal segment forms hook	MGI, DMDD
Sh3pxd2a$^{tm1b(EUCOMM)Wtsi}$/Sh3pxd2a$^{tm1b(EUCOMM)Wtsi}$	(C57BL/6N-Sh3pxd2a$^{tm1b(EUCOMM)Wtsi}$/Wtsi)	Sh3pxd2a	SH3 and PX domains 2A	8 out of 11	abnormal Mullerian duct morphology	J:239583	Müllerian duct	Left and right distal segment forms hook, left side segment forms a hook, right distal segment forms hook and left distal segment missing	MGI, DMDD
		Sh3pxd2a	SH3 and PX domains 2A	1 out of 11	abnormal Mullerian duct topology	J:239583	Müllerian duct	Left and right distal segment forms hook	MGI, DMDD
Slc25a20$^{tm1a(EUCOMM)Wtsi}$/Slc25a20$^{tm1a(EUCOMM)Wtsi}$	(C57BL/6N-Slc25a20$^{tm1a(EUCOMM)Wtsi}$/Wtsi)	Slc25a20	Solute carrier family 25 (mitochondrial carnitine/acylcarnitine translocase), member 20	1 out of 6	abnormal Mullerian duct morphology	J:239583	Müllerian duct	Left distal segment missing, Wolffian distal side is aberrant as well	MGI, DMDD
Sox9^{tm1Gsr}/Sox9^{tm1Gsr} ; Krt19$^{tm1(cre)Mmt}$/Krt19+	(involves: 129P2/OlaHsd * C57BL/6)	Sox9, Krt19	SRY (sex determining region Y)-box 9, keratin 19	No information	failure of Mullerian duct regression	J:104330	Müllerian duct	Absence of Mullerian duct regression in males	MGI, DMDD
Tcf7l2$^{tm1a(EUCOMM)Wtsi}$/Tcf7l2$^{tm1a(EUCOMM)Wtsi}$	(C57BL/6N-Tcf7l2$^{tm1a(EUCOMM)Wtsi}$/Wtsi)	Tcf7l2	Transcription factor 7 like 2	1 out of 5	abnormal Mullerian duct morphology	J:239583	Müllerian duct	Left, distal segment missing	MGI, DMDD
Tent5c$^{tm1b(KOMP)Wtsi}$/Tent5c$^{tm1b(KOMP)Wtsi}$	(C57BL/6N-Tent5c$^{tm1b(KOMP)Wtsi}$/Wtsi)	Tent5c	Terminal nucleotidyltransferase 5C	1 out of 8	abnormal Mullerian duct morphology	J:239583	Müllerian duct	Left and right distal segment forms hook	MGI, DMDD
Traf6$^{tm2a(EUCOMM)Wtsi}$/Traf6$^{tm2a(EUCOMM)Wtsi}$	(C57BL/6N-Traf6$^{tm2a(EUCOMM)Wtsi}$/Wtsi)	Traf6	TNF receptor-associated factor 6	1 out of 9	abnormal Mullerian duct morphology	J:239583	Müllerian duct	Right distal segment absent	MGI, DMDD
Wnt4^{tm1Amc}/Wnt4^{tm1Amc}	(involves: 129S1/Sv)	Wnt4	Wingless-type MMTV integration site family, member 4	No information	absent Mullerian ducts	J:52554	Müllerian duct	At E14.5, Mullerian duct development is absent in females and specific Mullerian cell markers (Wnt7a and Pax8) are not detected	MGI, DMDD
Wnt7a^{px}/Wnt7a^{px}	(involves: STOCK Sox18Ra)	Wnt7a	Wingless-type MMTV integration site family, member 7A	No information	abnormal Mullerian duct morphology	J:112	Müllerian duct	Anomalies of the Mullerian ducts in both sexes	MGI, DMDD
Wnt7a^{tm1Amc}/Wnt7a^{tm1Amc}	(involves: 129S1/Sv)	Wnt7a	Wingless-type MMTV integration site family, member 7A	No information	abnormal Mullerian duct morphology	J:171430, J:50342	Müllerian duct	Although Mullerian ducts are present neonatally, the Mullerian duct derivatives of mutant newborn and adult females fail to differentiate properly. Lumen of the Mullerian duct is expanded and the Mullerian duct mesenchyme is more condensed than in mice with conditional loss of Ctnnb1 in the Mullerian duct.	MGI, DMDD
		Wnt7a	Wingless-type MMTV integration site family, member 7A	No information	failure of Mullerian duct regression	J:171430, J:50342	Müllerian duct	Mutant males fail to undergo regression of the Mullerian duct due to absence of the receptor for Mullerian-inhibiting substance. Non-regressed Mullerian ducts appear as thin, undifferentiated tubes with no regional organization that run alongside the epididymis and the vas deferens from the testis to the urogenital sinus.	MGI, DMDD
Wnt9$^{btm1.1Amc}$/Wnt9b$^{tm1.1Amc}$	(involves: 129X1/SvJ * C57BL/6 * CBA)	Wnt9b	Wingless-type MMTV integration site family, member 9B	No information	abnormal Mullerian duct morphology	J:100575	Müllerian duct	Posterior extension of the Mullerian duct fails to occur	MGI, DMDD
Xpnpep1$^{tm1a(KOMP)Wtsi}$/Xpnpep1$^{tm1a(KOMP)Wtsi}$	(C57BL/6N-Xpnpep1$^{tm1a(KOMP)Wtsi}$/Wtsi)	Xpnpep1	X-prolyl aminopeptidase (aminopeptidase P) 1, soluble	2 out of 6	abnormal Mullerian duct morphology	J:239583	Müllerian duct	Right distal segment absent, left distal segment absent	MGI, DMDD
Zmynd11$^{em1(IMPC)Wtsi}$/Zmynd11$^{em1(IMPC)Wtsi}$	(C57BL/6N-Zmynd11$^{em1(IMPC)Wtsi}$/WtsiIeg)	Zmynd11	Zinc finger, MYND domain containing 11	1 out of 11	abnormal Mullerian duct topology	J:239583	Müllerian duct	Right distal segment forms a hook	MGI, DMDD

Allelic Composition	Genetic background	Gene Symbol	Gene name	Mutation penetrance	Annotated Term	Reference	Mutant phenotype	Further mutant phenotypical information	Database
Apc^{tm1Rsmi}/Apc^{tm1Rsmi} ; Pgr$^{tm2(cre)Lyd}$/Pgr+	(involves: 129P2/OlaHsd * 129S1/Sv * 129X1/SvJ * C57BL/6J)	Apc; Pgr	APC, WNT signaling pathway regulator; Progesterone receptor	No information	abnormal oviduct morphology	J:210095	Oviducts	87.2% tubular intraepithelial lesions in the epithelium of the distal oviduct and fimbriae. Loss of cilia, bulging of cells into the lumen, layering and suspicious stratification of cells, and rounding and hyperchromatization of the nucleus. 20% of mice develop glandular transformation of the normal papillary architecture of the oviduct characterized by glandular growth, loss of cilia, and general loss of normal cellular morphology.	MGI
Ar^{tm1Chc}/Ar$^{tm1.1Chc}$; Tmem163$^{Tg(ACTB-cre)2Mrt}$/0	(involves: 129S/SvEv * C57BL/6J * FVB/N)	Ar; Tmem163	Androgen receptor; Transmembrane protein 163	No information	decreased oviduct weight	J:87193	Oviducts	Oviducts from 4-, 6-, and 12-week old females weigh 18-23% less than those of control females	MGI
Brca1^{tm1Brn}/Brca1^{tm1Brn} ; Tg(Krt18-EGFP,-TAg121)36Ysng/0 ; Trp53^{tm1Brn}/Trp53^{tm2Tyj}	(involves: 129P2/OlaHsd * 129S4/SvJae * C57BL/6 * DBA/2 * FVB/N)	Brca1; Krt18; Trp53	Breast cancer 1; Keratin 18; Transformation related protein 53	No information	abnormal oviduct morphology	J:189304	Oviducts	2% of mice intrabursally injected with Ad-cre exhibit transformation of the oviduct epithelium, with lesions ranging from atypical hyperplasia, to carcinoma in situ to adenocarcinoma. Oviduct lesions are characterized by a glandular/acinar histology	MGI
Cbl^{tm1Wlan}/Cbl^{tm1Wlan}	(involves: 129S1/Sv * C57BL/6)	Cbl	Casitas B-lineage lymphoma	No information	dilated oviduct	J:81965	Oviducts	40% of females have dilated oviducts without hyperplasia possibly as a result of obstruction and mucous retention.	MGI
Dicer1^{tm1Bdh}/Dicer1^{tm1Bdh} ; Amhr2$^{tm3(cre)Bhr}$/Amhr2+	(involves: 129)	Amhr2 ; Dicer1	Anti-Mullerian hormone type 2 receptor ; Endoribonuclease Dicer	No information	abnormal oviduct morphology	J:142113	Oviducts	Lack of extensive coiling and appear more transparent under a dissecting microscope. Distended sac-like structures filled with clear fluid are observed at the end of the oviduct near the uterotubal junction. Oviducts of both immature and pregnant female mutants rupture easily, do not appear patent, and cannot be flushed. Mutant oviducts display a disorganized epithelial cell layer while the isthmus region is almost devoid of smooth muscle tissue.	MGI
					short oviduct	J:142113	Oviducts	Immature (day 25) and adult day-1 pregnant female mutants display shorter oviducts that are less than one half the length of control littermates	MGI
					abnormal oviduct transport	J:142113	Oviducts	Unlike embryos developing in wild-type females, embryos in mutant females fail to enter the uterus on day 4 of pregnancy, indicating disruption of oviductal transport; most are found in the upper one third of the oviduct instead of the uterus. Only a few embryos progress through the oviduct to the isthmus; however, these are mostly fragmented and some zonae pellucidae are lost. In contrast, in vitro cultured pronuclear (day-1) embryos collected from mutant donors develop at a similar rate as those derived from wild-type females.	MGI
Dicer1^{tm1Tara}/Dicer1^{tm1Tara} ; Pten^{tm1Hwu}/Pten^{tm1Hwu} ; Amhr2$^{tm3(cre)Bhr}$/Amhr2+	(involves: 129P2/OlaHsd * 129S4/SvJae * 129S7/SvEvBrd)	Dicer1; Pten; Amhr2	Endoribonuclease Dicer; Phosphatase And Tensin Homolog ; Anti-Mullerian hormone type 2 receptor	No information	abnormal oviduct morphology	J:182161	Oviducts	Abnormal proliferation begins in the stromal compartment, not in the epithelial layer, of the fallopian tube; tumor cells in the stroma express epithelial markers indicating that stromal cells in the fallopian tube undergo a transition to an epithelial cell type during carcinoma formation	MGI
Emx2^{tm1Sia}/Emx2^{tm1Sia}	(involves: C57BL/6 * CBA)	Emx2	Empty spiracles homeobox 2	No information	absent oviduct	J:40605	Oviducts	Mullerian ducts are absent at E13	MGI
Foxc1^{tm1Blh}/Foxc1^{tm1Blh}	(involves: 129S6/SvEvTac * Black Swiss)	Foxc1	Forkhead box C1	No information	abnormal oviduct morphology	J:105944	Oviducts	The oviduct is shorter than normal and uncoiled	MGI
		Foxc1	Forkhead box C1	No information	short oviduct	J:105944	Oviducts	The oviduct is shorter than normal and uncoiled	MGI
Foxj1^{tm1Bph}/Foxj1^{tm1Bph}	(involves: 129S1/Sv * 129X1/SvJ * Black Swiss)	Foxj1	Forkhead box J1	No information	absent oviduct epithelium motile cilium	J:50025	Oviducts	Homozygotes exhibit complete absence of cilia in the oviduct	MGI
Foxj1^{tm1Slb}/Foxj1^{tm1Slb}	(involves: 129X1/SvJ)	Foxj1	Forkhead box J1	No information	absent oviduct epithelium motile cilium	J:73616	Oviducts	Absence of classic motile type cilia with a 9 + 2 microtubule ultrastructure in the oviduct epithelial cells	MGI
Hoxa$^{tm1.1Ssp}$/Hoxa$^{tm1.1Ssp}$	involves: 129 * CD-1	Hoxa9,10,11	Homeobox A cluster	No information	abnormal female reproductive system morphology	J:198638	Oviducts	Severe anterior homeotic transformations of reproductive structures. The uterotubal junction (connecting the uterus and oviduct) is poorly defined, elongated and shifted posteriorly. Uteri resemble oviducts	MGI
Kif19a^{tm1Noh}/Kif19a+	(involves: 129S6/SvEvTac * C57BL/6J)	Kif19a	Kinesin family member 19A	No information	abnormal oviduct epithelium motile cilium morphology	J:191072	Oviducts	1.4 fold increase in cilia length	MGI
Kif19a^{tm1Noh}/Kif19at^{m1Noh}	(involves: 129S6/SvEvTac * C57BL/6J)	Kif19a	Kinesin family member 19A	No information	abnormal oviduct epithelium motile cilium morphology	J:191072	Oviducts	2.5 fold increase in cilia length	MGI
		Kif19a	Kinesin family member 19A	No information	abnormal oviduct morphology	J:191072	Oviducts	Lumens are filled with mucus and debris. Debris contains nuclear structures suggesting it is made up of dead cells	MGI
		Kif19a	Kinesin family member 19A	No information	abnormal female reproductive system physiology	J:191072	Oviducts	Movement of oviduct ciliary tips is abnormal, disorganized, and fails to generate proper fluid flow	MGI
Mir34a$^{tm1.1Pisc}$/Mir34a$^{tm1.1Pisc}$; Mirc21$^{tm1.1Pisc}$/Mirc21$^{tm1.1Pisc}$; Mirc34$^{tm1.1Pisc}$/Mirc34$^{tm1.1Pisc}$	(involves: 129S4/SvJae * C57BL/6 * C57BL/6J)	Mir34a, Mirc21, Mirc34	MicroRNA 34a; MicroRNA cluster 21; MicroRNA cluster 34	No information	abnormal oviduct epithelium motile cilium morphology	J:256929	Oviducts	Strong reduction in the number of multicilia	MGI
		Mir34a, Mirc21, Mirc34	MicroRNA 34a; MicroRNA cluster 21; MicroRNA cluster 34	No information	abnormal oviduct transport	J:256929	Oviducts	2.5 days after superovulation oocytes remain trapped in the bursa of the ovary	MGI
		Mir34a, Mirc21, Mirc34	MicroRNA 34a; MicroRNA cluster 21; MicroRNA cluster 34	No information	abnormal oviduct physiology	J:256929	Oviducts	Increase in the fraction of proliferating cells in the epithelium, increase in proliferating cells is inversely correlated with the decrease in ciliation	MGI
Odf2$^{tm1.1Sats}$/Odf2$^{tm1.2Sats}$; Tg(CAG-cre)13Miya/0	(involves: C57BL/6 * C57BL/6J)	Odf2	Outer dense fiber of sperm tails 2	No information	abnormal oviduct epithelium motile cilium morphology	J:181294	Oviducts	Tendency toward a decrease in the number of cilia per cell in multiciliated cells, in cilia basal feet are absent	MGI
Pax2^{tm1Mbu}/Pax2^{tm1Mbu} ; (C3.129P2-Pax2^{tm1Mbu})	(C3.129P2-Pax2^{tm1Mbu})	Pax2	Paired box 2	No information	absent oviduct	J:63683	Oviducts	At E18.5, female homozygotes lack oviducts	MGI
Pax2^{tm1Pgr}/Pax2^{tm1Pgr}	(involves: 129S1/Sv * 129X1/SvJ)	Pax2	Paired box 2	No information	absent oviduct	J:30343	Oviducts	Absent oviduct	MGI
Pax8^{tm1Pgr}/Pax8^{tm1Pgr}	(either: (involves: 129S1/Sv * 129X1/SvJ) or (involves: 129S1/Sv * 129X1/SvJ * C57BL/6))	Pax8	Paired box 8	No information	dilated oviduct	J:129545	Oviducts	82% of T4-substituted female homozygotes develop a dilated, fluid-filled fallopian tube (hydrosalpinx) with a flattened mucosal layer within 6 months of age	MGI
Postn^{tm1Sjc}/Postn^{tm1Sjc}	(involves: C57BL/6)	Postn	Periostin, osteoblast specific factor	No information	small oviduct	J:103753	Oviducts	Smaller, in proportion with overall reduction in body size. Histology appears normal.	MGI

Prlr^{tm1Cnp}/Prlr^{tm1Cnp}	(either: (involves: 129/Sv * 129P2/OlaHsd) or (involves: 129P2/OlaHsd * C57BL/6))	Prlr	Prolactin receptor	No information		abnormal oviduct environment	J:38093	Oviducts	Defective pre-implantation embryonic development	MGI
Pten^{tm1Hwu}/Pten$^{tm1Hwu\,;}$ Vhl^{tm1Jae}/Vhl^{tm1Jae} ; Tg(Cdh16-cre)91Igr/0	(involves: 129S4/SvJae * ICR)	Pten; Vhl; Cdh16	Phosphatase and tensin homolog; Von Hippel-Lindau tumor suppressor; Cadherin 16	No information		abnormal oviduct morphology	J:137442	Oviducts	Oviduct abnormalities	MGI
Rara^{tm1Ipc}/Rara$^{tm1Ipc\,;}$ Rarb^{tm1Ipc}/Rarb^{tm1Ipc}	(involves: 129S2/SvPas)	Rara, Rarb	Retinoic acid receptor, alpha, Retinoic acid receptor, beta		1	absent oviduct	J:21034	Oviducts	At E18.5 the uterine tubes, uterus and the cranial vagina that are all derived from the paramesonephric ducts are absent due to the absence of these ducts (6 out of 6)	MGI
Rara^{tm1Ipc}/Rara^{tm1Ipc} ; Rarb^{tm1Mma}/Rarb^{tm1Mma}	(involves: 129S2/SvPas * C57BL/6)	Rara, Rarb	Retinoic acid receptor, alpha, Retinoic acid receptor, beta		1	absent oviduct	J:43344	Oviducts	100% penetrance of agenesis of oviducts	MGI
Safb^{tm1So}/Safb^{tm1So}	(involves: 129 * C57BL/6J)	Safb	Scaffold attachment factor B	No information		oviduct atrophy	J:97633	Oviducts	Females that failed to become pregnant 4 out of 6 had smaller oviducts that showed atrophy with increasing age	MGI
		Safb	Scaffold attachment factor B	No information		small oviduct	J:97633	Oviducts	Females that failed to become pregnant 4 out of 6 had a thinner muscle layer around the oviduct and the oviducts were disproportionately smaller than normal	MGI
		Safb	Scaffold attachment factor B	No information		Abnormal oviduct transport	J:97633	Oviducts	In females that failed to become pregnant at 3.5 days post conception embryos are still found in the oviducts rather than in the uterus as in wild-type females	MGI
sho/sho	(Not Specified)	Sho	Shorthead	No information		abnormal oviduct morphology	J:255	Oviducts	One may occur medially to kidney	MGI
Trp63^{tm1Fmc}/Trp63^{tm1Fmc}	(involves: 129S4/SvJae)	Trp63	Transformation related protein 63	No information		absent oviduct	J:79340	Oviducts	Absent oviduct	MGI
Vgf^{tm1Srjs}/Vgf^{tm1Srjs}	(involves: 129S1/Sv * 129X1/SvJ * C57BL/6)	Vgf	VGF nerve growth factor inducible	No information		decreased oviduct weight	J:56499	Oviducts	Oviducts weighed 18-30% less the weight of those found in wild-type mic	MGI
Wnt7a^{tm1Amc}/Wnt7a^{tm1Amc}	(involves: 129S1/Sv)	Wnt7a	Wingless-type MMTV integration site family, member 7A	No information		abnormal oviduct morphology	J:50342	Oviducts	The anterior regions of the mutant reproductive tract display a fimbriated, ciliated epithelium typical of the proximal oviduct while posterior regions show a less elaborately folded mucosa composed of a simple columnar epithelium which resembles the isthmic region of the oviduct. Although regional differentiation occurs along the oviduct, normal elongation and coiling of the oviduct fail to occur. No visibly coiled oviducts are present in newborn and adult female mutants.	MGI
		Wnt7a	Wingless-type MMTV integration site family, member 7A	No information		short oviduct	J:50342	Oviducts	Mutant oviducts are shortened and uncoiled	MGI
Wnt9b$^{tm1.1Amc}$/Wnt9b$^{tm1.1Amc}$	(involves: 129X1/SvJ * C57BL/6 * CBA)	Wnt9b	Wingless-type MMTV integration site family, member 9B	No information		oviduct hypoplasia	J:100575	Oviducts	Most of the oviduct is absent at birth	MGI
